# Effects of a randomized controlled trial of unconditional cash transfers on epigenetic measures of ageing and cognition in children and mothers

**DOI:** 10.1038/s41562-026-02568-4

**Published:** 2026-09-08

**Authors:** Laurel Raffington, Yayouk E. Willems, Jessica F. Sperber, Sepideh Zarandooz, Molly A. Costanzo, Daniel A. Notterman, Colter Mitchell, Elliot M. Tucker-Drob, Greg J. Duncan, Nathan A. Fox, Lisa A. Gennetian, Katherine A. Magnuson, Sonya V. Troller-Renfree, Hirokazu Yoshikawa, Elisabeth B. Binder, K. Paige Harden, Kimberly G. Noble

**Affiliations:** 1 Max Planck Research Group Biosocial—Biology, Social Disparities, and Development, Max Planck Institute for Human Development, Berlin, Germany; 2 Teachers College, Columbia University, New York City, NY USA; 3 Institute for Research on Poverty, University of Wisconsin–Madison, Madison, WI USA; 4 Department of Molecular Biology, Princeton University, Princeton, NJ USA; 5 Survey Research Center, University of Michigan, Ann Arbor, MI USA; 6 Population Studies Center, University of Michigan, Ann Arbor, MI USA; 7 Department of Psychology, University of Texas at Austin, Austin, TX USA; 8 Population Research Center, University of Texas at Austin, Austin, TX USA; 9 School of Education, University of California, Irvine, Irvine, CA USA; 10 Department of Human Development and Quantitative Methodology, University of Maryland, College Park, MD USA; 11 Sanford School of Public Policy, Duke University, Durham, NC USA; 12 Global TIES for Children Center, New York University, New York City, NY USA; 13 Department Genes and Environments, Max Planck Institute of Psychiatry, Munich, Germany

**Keywords:** Development studies, DNA methylation, Economics

## Abstract

Here we present epigenetic findings from a randomized controlled trial studying the impact of unconditional cash gifts of US$333 per month (children *n* = 313; mothers *n* = 329) or US$20 per month (children *n* = 423; mothers *n* = 448) to US mothers with low income during their child’s first four years of life. Salivary DunedinPACE-pace of aging, a DNA-methylation predictor of healthy lifespan, and Epigenetic-*g*, a DNA-methylation biomarker of cognition, were preregistered as secondary outcomes (clinical trial identifier NCT03593356). As hypothesized, high-cash compared with low-cash gifts caused a −0.17 s.d. (95% confidence interval −0.32 to −0.01, *P* = 0.037) difference in children’s DunedinPACE. We found no evidence supporting our hypothesis that higher cash gifts increased children’s Epigenetic-*g*, although it did correlate with children’s brain activity. There was no evidence that the cash gift had an impact on these epigenetic indices in mothers. These findings show a detectable impact of monthly unconditional cash received through early childhood on epigenetic indices of 4-year-old children in families with low income. Whether this epigenetic effect persists and how it may relate to behavioural and health outcomes remains unknown.

## Abstract (web summary)

Raffington et al. find that providing low-income mothers with cash gifts for the first four years of their children’s lives appears to change the children’s epigenetic biological ageing in ways associated with better health in adulthood.

## Main

Mothers and children who live in poverty experience more frequent illnesses, earlier onset of diseases, greater incidence of chronic health conditions, higher rates of obesity, poorer cognitive function, poorer mental health and shorter lifespan, compared with their more affluent counterparts^1–7^. The first five years of life appears to be an especially sensitive period during which low income may have the largest negative impacts on subsequent health^2,8,9^. However, identifying the causal effect of income on health is difficult, because family income is correlated with numerous factors, and because cash transfer study designs are complex and time-consuming^10–12^. The Baby’s First Years (BFY) randomized controlled trial was designed to identify the causal impact of increased income on maternal and child health, cognition, behaviour and brain development among families with low income in the USA^13^. Beginning shortly after birth and continuing for 6 years, low-income mothers residing in the USA received unconditional monthly cash gifts of either US$333 per month or US$20 per month. While the core BFY framework emphasizes family investment and family stress pathways, the present study examines a complementary biological pathway that may link family income to child development and adult health.

Previous research found that the BFY high-cash gift has selective positive impacts on early childhood investments and some aspects of infant brain development, but few of the hypothesized effects on maternal and child health have been observed. Specifically, the high-cash gift increased household spending on early childhood enrichment items such as toys and books, as well as increased maternal engagement in activities such as reading with the BFY focal child, and mothers reported increased consumption of fresh produce among toddlers^14–17^. Infants in the high-cash versus low-cash gift group showed suggestive, non-preregistered evidence of more high-frequency brain activity and more alpha power at 4 years old^18,19^. However, the high-cash gift had no observed impact on numerous other family and maternal measures including material hardship, a preregistered composite of fast-paced brain activity, food insecurity, or maternal physical or psychological well-being. There were also no impacts on maternal reports of child health, language and socioemotional skills, or direct assessments of child executive function and vocabulary^14,15,17,20^. These null findings for BFY focal children’s outcomes might indicate that unconditional cash does not affect these outcomes, that the modest income boost was not enough to overcome other challenges linked to poverty, or—as has been found for other childhood interventions^21^—that the benefits of increased income during early childhood among children residing in poverty may not be evident until later in life.

One hypothesized biological pathway through which early life economic circumstances might impact later-life health and cognition—before observable behavioural differences—is through the epigenome^22,23^. Epigenetic modifications, such as DNA methylation (DNAm), involve specific molecular changes that regulate gene expression and phenotypic plasticity^24^. These modifications can persist through a lifetime, but are also subject to change in response to environmental, behavioural and age-related factors^25^. The BFY study provides a unique opportunity to test whether income boosts through a monthly unconditional cash gift to families experiencing poverty affect the human epigenome.

Recently, DNAm algorithms known as ‘epigenetic clocks’^25^ have emerged as among the most widely studied and best-validated predictors of physical health and mortality in adulthood^26–29^. Although epigenetic clocks were predominantly trained using data from White adults in midlife and beyond, studies in racially and socioeconomically diverse adults provide evidence for the generalizability of epigenetic ageing clocks^30,31^. Accordingly, epigenetic clocks have received consensus support for use as outcomes in intervention studies of ageing^32^. Trajectories of epigenetic ageing are hypothesized to commence very early in development, decades before the onset of ageing-related disease^22,33,34^, although the interpretation of clock measures in children is not resolved.

Children’s epigenetic clocks are correlated with family income and other indicators of socioeconomic status (SES). A meta-analysis of 140 studies found that childhood SES was most strongly correlated with specific epigenetic clocks, which were developed to quantify the pace of biological ageing using longitudinal data on long-term declines in organ-system integrity^35^. In adults, epigenetic pace of ageing prospectively predicts chronic disease onset and mortality up to 20 years later and can be modified by health-promoting interventions^36,37^. In two US-based cohorts spanning late childhood through adolescence, epigenetic pace of ageing has been found to correlate with children’s body mass index (BMI), pubertal timing and mental health problems^7,38,39^. Epigenetic pace of ageing, therefore, appears as a promising metric to track the biological embedding of health risks early in life^22,40^. So far, it has not been used as an outcome in studies of early childhood interventions that aim to improve later health.

A related epigenetic algorithm, ‘Epigenetic-*g*’, attempts to quantify ageing-related cognitive differences using DNAm patterns. Epigenetic-*g* was trained to predict performance on multiple cognitive tests in a cohort of Scottish adults^41^. In separate samples of older adults, including a racially and sociodemographically diverse US cohort, Epigenetic-*g* has been associated with circulating levels of neurology- and inflammation-related proteins, brain cortical volumes, cognitive ageing and 6-year risk of dementia^41,42^. Epigenetic-*g* has received much less research attention than epigenetic clocks, but two studies in children and adolescents found that salivary Epigenetic-*g* was distinct from epigenetic pace of ageing, and correlated in expected directions with cognitive test performance and family SES^43,44^. As with the epigenetic clocks, Epigenetic-*g* has not yet been integrated in studies testing the causal impact of early childhood environments.

Here, we use data from the BFY study to investigate whether mothers and their 4-year-old children in families receiving a larger monthly cash gift had differing epigenetic indices than those receiving a lower monthly cash gift. Before age-4 data collection, we designated DNAm measures of epigenetic pace of ageing (DunedinPACE) and Epigenetic-*g* as secondary outcomes of interest in both children and mothers (clinical trial identifier NCT03593356). After data collection concluded but before generating epigenetic data, we publicly preregistered a detailed analysis plan (https://osf.io/8grhj). We hypothesized that children and mothers in the high-cash gift group would have lower DunedinPACE and higher Epigenetic-*g* compared with those in the low-cash gift group. For supplemental preregistered epigenetic outcomes, we examined biological age acceleration as measured by PhenoAge^45^ and GrimAge^46^; based on prior research in children^35,39^, we preregistered null effects, but report them given their widespread use in epigenetic clock research. Given the richness of BFY data measuring a variety of children’s developmental outcomes, we also preregistered analyses examining associations between epigenetic measures and concurrent measures of children’s brain activity, cognition and health.

## Results

### The impact of the high-cash gift on children’s epigenetic indices

**Fig. 1:**
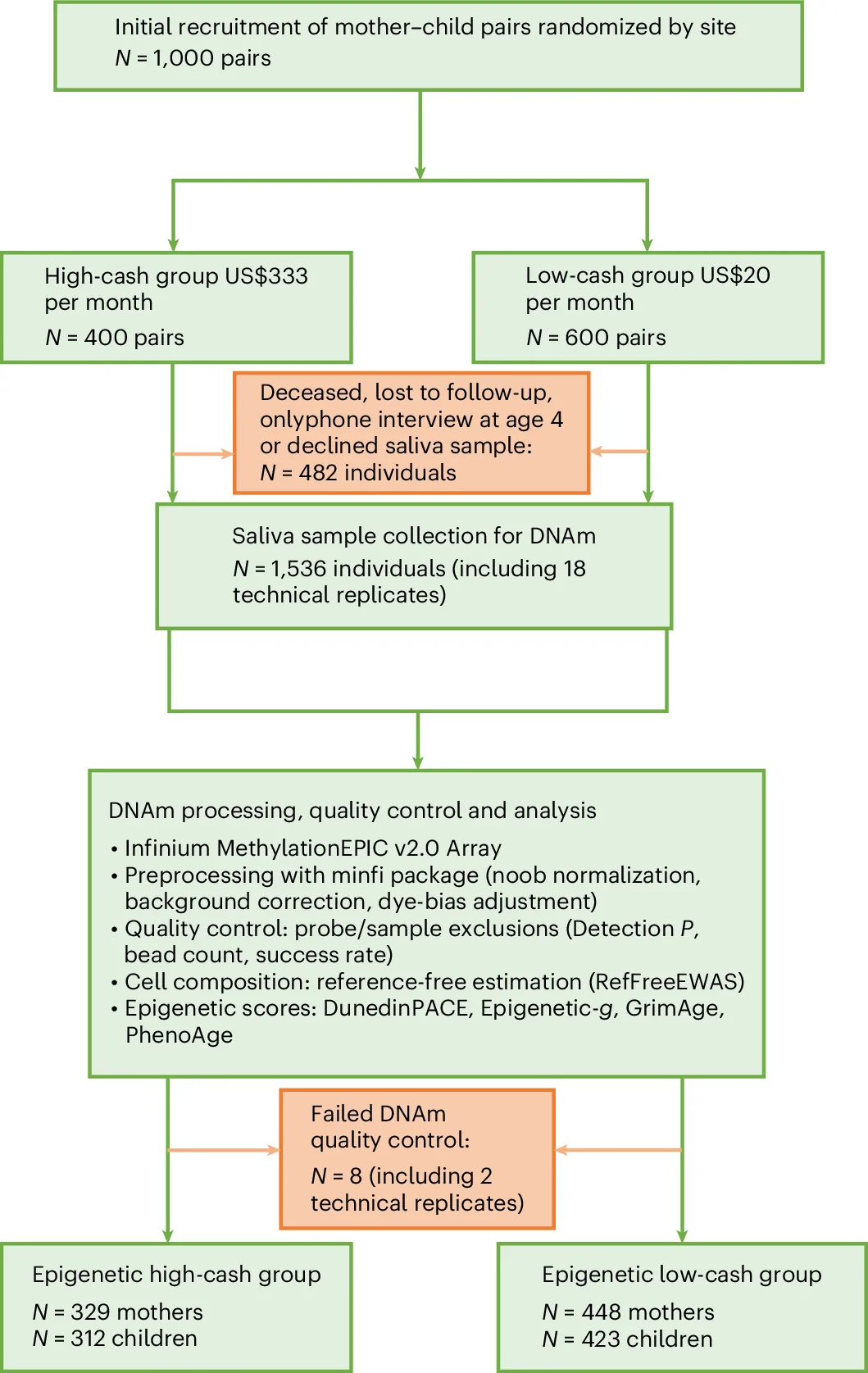
CONSORT diagram. The CONSORT diagram details participant flow through enrolment, randomization, follow-up and analysis.

We estimated the impact of a cash gift of US$313 per month, that is, the difference between the high-cash and low-cash gift group, using intent-to-treat (ITT) models (Table 1). These analyses included preregistered pretreatment baseline covariates: maternal characteristics, household characteristics, birth characteristics of the infant, and the child’s sex. Figure 1 shows a CONSORT diagram that details participant flow through enrolment, randomization, follow-up and analysis. Measures of DNAm were available from 735 BFY focal children’s saliva at age 4. Supplementary Tables 1 and 2 show that there was no evidence of treatment group differences across a set of baseline covariates (joint test *P* level: 0.23). Supplementary Table 3 reports a correlation matrix of child epigenetic indices and baseline covariates. Epigenetic indices were adjusted for cell composition. There was no evidence of differences in cell composition, particularly the distribution of immune cells, between high- and low-cash gift groups for children or mothers (children: standardized (std.) *β* = 0.006, standard error (SE) = 0.014, *P* = 0.66; mothers: std. *β* = 0.001, SE = 0.013, *P* = 0.95).

**Table 1:** Summary of the estimated impacts of the high-cash gift from ITT models assessing differences in children’s epigenetic indices of biological ageing and cognition in high-cash and low-cash gift groups (*N* = 735)

	(1)	(2)	(3)	(4)	(5)	(6)
	Low-cash gift group mean	High-cash gift group mean	OLS with site fixed effects	OLS with baseline covariates	Effect size (*d*)	Two-tailed *P* value
DunedinPACE	0.005	−0.006	−0.011* (0.005) [−0.02 to 0.00]	−0.011* (0.005) [−0.02 to 0.00]	−0.17*	0.037*
Epigenetic-*g*_separate	0.005	−0.007	−0.012 (0.007) [−0.03 to 0.00]	−0.014 (0.008) [−0.03 to 0.00]	−0.15	0.077
Epigenetic-*g*_across	0.006	−0.009	−0.015* (0.006) [−0.03 to 0.00]	−0.016* (0.006) [−0.03 to 0.00]	−0.19*	0.016*
GrimAge Accel	−0.015	0.020	0.035 (0.058) [−0.07 to 0.15]	0.038 (0.060) [−0.08 to 0.16]	0.05	0.529^a^
PhenoAge Accel	−0.075	0.102	0.174 (0.132) [−0.08 to 0.43]	0.175 (0.135) [−0.09 to 0.44]	0.10	0.392^a^
Adjusts for data collection site			X	X	X	X
Adjusts for baseline covariates				X	X	X

Robust standard errors in parentheses, 95% CIs in brackets. Epigenetic profiles were residualized for technical factors before analyses (cell type composition, slide and array). Column 3 includes fixed effects for the data collection site. Column 4 introduces covariates for child age (in months) at the age-4 visit, fixed effects for the interviewer that conducted the data collection and the following characteristics from the baseline survey: maternal characteristics (age, education, race and ethnicity, marital status, general health, depressive symptoms and cigarette and alcohol consumption during pregnancy), household characteristics (number of children born to mother, number of adults in the household, father living with the mother, household income and household net worth) and infant birth characteristics (weight at birth and gestational age). *P < 0.05.

^a^We grouped the analysis of PhenoAge and GrimAge Acceleration in the same family of tests, because they are both tapping into the construct of ‘accelerated biological ageing’, and report FDR-adjusted *P* values to correct for multiple testing.

Results show that the higher cash gifts slowed children’s epigenetic pace of biological ageing, as measured by DunedinPACE (−0.17 s.d. difference between high- and low-cash gift groups, 95% confidence interval (CI) −0.32 to −0.01, *P* = 0.037; Table 1). DunedinPACE treatment group differences were robust to sensitivity checks, including the addition of non-response weights adjusting for differential attrition due to available DNAm (*P* = 0.023, *P* = 0.032; Supplementary Table 4). As hypothesized, we found no evidence that the other epigenetic clocks that we examined—GrimAge and PhenoAge Acceleration measures—differed between the high- and low-cash gift groups (Table 1).

Results for Epigenetic-*g* were more ambiguous. Unlike DunedinPACE, the calculation of Epigenetic-*g* includes a scaling function that normalizes DNAm at individual DNAm sites across people within the sample. This procedure can account for technical factors in DNAm batches. However, DNAm varies with age, and in samples with a wide age range, such as a pooled sample of mothers and their children, this procedure normalizes DNAm values relative to the total variance, compressing variation among similarly aged people. Because the original algorithm and our preregistered analysis plan lacked specificity about whether to compute Epigenetic-*g* separately in mothers and children (‘Epigenetic-*g*_separate’) or across maternal and child DNAm samples together (‘Epigenetic-*g*_across’), we considered this to be an important sensitivity check. In child DNAm, Epigenetic-*g_*separate and Epigenetic-*g_*across were nearly perfectly correlated (*r* = 0.97, 95% CI 0.95 to 0.99), but the range and variance of Epigenetic-*g*_across was slightly smaller than Epigenetic-*g*_separate in children and in mothers. Epigenetic-*g*_separate is our preferred specification, because its variance is not reduced by age-related DNAm differences between mothers and their child.

For Epigenetic-*g_*separate, the ITT impact estimate was in the opposite direction of our hypothesized outcome (lower Epigenetic-*g* in children from the high-cash treatment group), but was not different from zero (−0.15 s.d., 95% CI −0.31 to 0.02, *P* = 0.077; Table 1). For Epigenetic-*g_*across, the ITT effect size was similar in direction and magnitude as Epigenetic-*g*_separate and was significantly different from zero (−0.19 s.d., 95% CI −0.35 to −0.04, *P* = 0.016). However, this effect was no longer statistically significant in one of two sensitivity checks that use weights to correct for non-response and covariate imbalance between groups (*P* = 0.053, *P* = 0.021; Supplementary Table 4). Overall, we found no evidence to support our preregistered directional hypothesis that the high-cash gift increased children’s Epigenetic-*g*.

### Associations among children’s epigenetic indices and children’s brain activity, cognition and health

Following our preregistration, we next explored how children’s epigenetic indices are related to each other and to concurrent measures of their brain activity, cognition and health pooling data from BFY focal children in the high- and low-cash gift group. DunedinPACE and Epigenetic-*g* were negatively correlated with each other, regardless of specification, but again, the significance at threshold *P* < 0.05 differed between Epigenetic-*g_*separate (*r* = −0.06) and Epigenetic-*g_*across (*r* = −0.10; Table 2). DunedinPACE was not correlated with relative electroencephalography (EEG) power in any of the four frequency bands measured (theta, alpha, beta or gamma) (*r* range from −0.04 to 0.03; Table 3). Correlations with absolute power are presented in Supplementary Table 5. DunedinPACE was also not significantly correlated with child executive functioning, receptive vocabulary, BMI or maternal reports of overall health (*r* range from −0.07 to 0.02; Table 3).

**Table 2:** Correlation matrix of child and maternal epigenetic indices and chronological age

	(1)	(2)	(3)	(4)	(5)	(6)	(7)	(8)	(9)	(10)	(11)
(1) Child chronological age^a^	1.00										
(2) Child DunedinPACE	−0.03 (0.41)	1.00									
	[−0.10 to 0.04]										
(3) Child Epigenetic-*g*_ across	0.00	−0.06	1.00								
	(0.99) [−0.07 to 0.07]	(0.09) [−0.14 to 0.01]									
(4) Child Epigenetic-*g*_ separate	0.01 (0.83)	−0.10* (0.01)	0.97* (0.00)	1.00							
	[−0.06 to 0.08]	[−0.18 to −0.03]	[0.97 to 0.97]								
(5) Child PhenoAge Accel	0.00	0.11*	0.01	0.04	1.00						
	(1.00) [−0.07 to 0.07]	(0.00) [0.04 to 0.18]	(0.73) [−0.06 to 0.08]	(0.33) [−0.04 to 0.11]							
(6) Child GrimAge Accel	0.00	0.18*	−0.04	−0.04	0.51*	1.00					
	(1.00) [−0.07 to 0.07]	(0.00) [0.11 to 0.25]	(0.33) [−0.11 to 0.04]	(0.27) [−0.11 to 0.03]	(0.00) [0.45 to 0.56]						
(7) Maternal chronological age	−0.02	0.03	−0.02	−0.01	−0.01	−0.04	1.00				
	(0.51) [−0.07 to 0.07]	(0.38) [−0.04 to 0.11]	(0.61) [−0.09 to 0.05]	(0.78) [−0.08 to 0.06]	(0.78) [−0.08 to 0.07]	(0.26) [−0.11 to 0.03]					
(8) Maternal DunedinPACE	−0.07	0.08*	0.00	−0.04	0.04	0.02	0.14*	1.00			
	(0.06) [−0.15 to 0.00]	(0.03) [0.01 to 0.15]	(0.92) [−0.07 to 0.08]	(0.33) [−0.11 to 0.04]	(0.24) [−0.03 to 0.11]	(0.59) [−0.06 to 0.09]	(0.00) [0.06 to 0.20]				
9) Maternal Epigenetic-*g*_ across^a^	0.03 (0.33) [−0.05 to 0.10]	0.02 (0.63) [−0.06 to 0.09]	0.21* (0.00) [0.14 to 0.28]	0.20* (0.00) [0.13 to 0.27]	−0.06 (0.09) [−0.13 to 0.01]	−0.07 (0.07) [−0.14 to 0.01]	0.27* (0.00) [0.21 to 0.34]	0.04 (0.29) [−0.04 to 0.12]	1.00		
(10) Maternal Epigenetic-*g*_ separate^a^	0.03	0.02	0.20*	0.19*	−0.05	−0.06	0.31*	0.05	0.98*	1.00	
	(0.40)	(0.52)	(0.00)	(0.00)	(0.14)	(0.14)	(0.00)	(0.15)	(0.00)		
	[−0.05 to 0.10]	[−0.05 to 0.10]	[0.12 to 0.26]	[0.12 to 0.26]	[−0.13 to 0.02]	[−0.13 to 0.02]	[0.24 to 0.37]	[−0.03 to 0.12]	[0.98 to 0.98]		
(11) Maternal PhenoAge Accel	−0.06	0.00	0.08*	0.08*	0.14*	0.01	0.00	0.17*	0.11*	0.11*	1.00
	(0.12) [−0.12 to 0.02]	(0.94) [−0.08 to 0.07]	(0.03) [0.01 to 0.15]	(0.02) [0.01 to 0.16]	(0.00) [0.07 to 0.22]	(0.77) [−0.06 to 0.08]	(1.00) [−0.06 to 0.08]	(0.00) [0.12 to 0.26]	(0.00) [0.04 to 0.18]	(0.00) [0.04 to 0.18]	
(12) Maternal GrimAge Accel	0.04	−0.02	0.05	0.04	0.14*	0.26*	0.00	0.19*	0.02	0.05	0.25*
	(0.31)	(0.67)	(0.21)	(0.24)	(0.00)	(0.00)	(1.00)	(0.00)	(0.67)	(0.14)	(0.00)
	[−0.09 to 0.06]	[−0.09 to 0.06]	[−0.03 to 0.12]	[−0.03 to 0.12]	[0.07 to 0.21]	[0.19 to 0.33]	[−0.07 to 0.08]	[0.13 to 0.27]	[−0.07 to 0.08]	[−0.03 to 0.12]	[0.17 to 0.31]

*P* values in parentheses, 95% CIs in brackets. ^a^There is no variation in children’s chronological age in years, only months. *P < 0.05.

**Table 3:** Associations between children’s epigenetic clocks and developmental traits at age 4

Outcomes	Dunedin PACE	*P* value	Epigenetic-*g*_separate	*P* value	Epigenetic-*g_* across	*P* value	PhenoAge Accel	*P* value	GrimAge Accel	*P* value	*N*
BMI	−0.02 (0.04) [−0.09 to 0.05]	0.593	−0.04 (0.04) [−0.11 to 0.03]	0.268	−0.04 (0.04) [−0.11 to 0.03]	0.286	0.00 (0.04) [−0.07 to 0.07]	0.992	0.06 (0.04) [−0.01 to 0.13]	0.081	713
Overall health	−0.05 (0.04) [−0.13 to 0.02]	0.162	−0.05 (0.03) [−0.12 to 0.02]	0.150	−0.05 (0.03) [−0.11 to 0.02]	0.189	−0.02 (0.04) [−0.09 to 0.06]	0.669	−0.01 (0.04) [−0.08 to 0.07]	0.838	732
Executive function	0.00 (0.04) [−0.08 to 0.08]	0.980	0.02 (0.04) [−0.06 to 0.10]	0.628	0.02 (0.04) [−0.06 to 0.10]	0.628	−0.06 (0.04) [−0.14 to 0.01]	0.080	−0.04 (0.04) [−0.11 to 0.03]	0.286	690
Receptive vocabulary monolinguals	−0.04 (0.05) [−0.14 to 0.06]	0.406	0.02 (0.05) [−0.08 to 0.13]	0.650	0.03 (0.05) [−0.07 to 0.14]	0.558	−0.02 (0.04) [−0.11 to 0.06]	0.588	−0.08 (0.04) [−0.17 to 0.01]	0.069	373
Receptive vocabulary bilinguals	−0.07 (0.07) [−0.21 to 0.07]	0.331	0.05 (0.07) [−0.08 to 0.19]	0.442	0.06 (0.07) [−0.08 to 0.19]	0.426	−0.01 (0.08) [−0.17 to 0.16]	0.950	−0.07 (0.07) [−0.21 to 0.07]	0.308	251
Total behaviour problems^a^	−0.01 (0.04) [−0.08 to 0.06]	0.762	0.05 (0.04) [−0.02 to 0.12]	0.186	0.05 (0.04) [−0.03 to 0.12]	0.202	0.02 (0.04) [−0.05 to 0.09]	0.609	0.00 (0.04) [−0.07 to 0.07]	0.924	731
Attention/impulsivity^a^	0.02 (0.04) [−0.05 to 0.10]	0.556	0.00 (0.03) [−0.07 to 0.06]	0.887	0.00 (0.03) [−0.07 to 0.06]	0.930	−0.06 (0.04) [−0.14 to 0.01]	0.091	−0.12* (0.04) [−0.20 to −0.05]	0.001	735
Relative whole brain power^b^											
Theta	0.02 (0.04) [−0.06 to 0.11]	0.582	0.01 (0.04) [−0.07 to 0.10]	0.734	0.02 (0.04) [−0.07 to 0.10]	0.687	−0.08 (0.05) [−0.18 to 0.02]	0.122	−0.05 (0.05) [−0.14 to 0.04]	0.317	588
Alpha	−0.04 (0.04) [−0.12 to 0.04]	0.335	0.04 (0.04) [−0.04 to 0.12]	0.313	0.04 (0.04) [−0.04 to 0.12]	0.318	0.08 (0.05) [−0.01 to 0.17]	0.093	0.02 (0.04) [−0.07 to 0.10]	0.716	588
Beta	0.01 (0.04) [−0.06 to 0.08]	0.762	−0.08* (0.04) [−0.16 to 0.00]	0.047	−0.09* (0.04) [−0.17 to −0.01]	0.035	0.04 (0.04) [−0.05 to 0.12]	0.416	0.05 (0.04) [−0.03 to 0.13]	0.224	588
Gamma	0.03 (0.04) [−0.04 to 0.10]	0.376	−0.08* (0.04) [−0.16 to 0.00]	0.045	−0.08* (0.04) [−0.16 to 0.00]	0.038	−0.02 (0.04) [−0.10 to 0.07]	0.726	0.05 (0.04) [−0.02 to 0.13]	0.172	588

Robust standard errors in parentheses, 95% CIs in brackets. Child models adjust for child sex and age at the time of the assessment (in months). All variables were standardized to the full sample before analyses, so coefficients can be interpreted as standardized betas. Epigenetic indices were residualized for technical factors before regression analyses (cell composition, slide and array). ^a^Added as a non-preregistered outcome. ^b^Brain activity models further adjust for the number of useable EEG epochs and are restricted to those with sufficient data. *P < 0.05.

For both Epigenetic-*g* specifications, lower Epigenetic-*g* was significantly correlated with higher relative EEG power in the beta and gamma bands (*r* range from −0.08 to −0.09; Table 3), which, in turn, were weakly associated with worse parent-reported attention problems at age 4 in a previous BFY study^18^. Due to these associations of EEG power with attention, we performed additional non-preregistered analyses examining associations between epigenetic measures and maternal reports of total behaviour problems, as captured via the Child Behavior Checklist (CBCL), as well as attention/impulsivity issues as rated by a research assistant using the Preschool Self-Regulation Assessment (PSRA). There was no evidence that Epigenetic-*g* was associated with reports of attention, executive functioning or receptive vocabulary (*r* range from −0.05 to 0.06; Table 3).

We also explored whether correlations between epigenetic indices and other child outcomes differed by cash gift group. For DunedinPACE and Epigenetic-*g*, they did not (Supplementary Table 6). When examining outcome measures of accelerated biological age, we found a treatment group by GrimAge Acceleration interaction on overall health (*P* = 0.030; Supplementary Table 7). This result suggests that, in the high-cash gift group (std. *β* = −0.13, SE 0.06, *P* = 0.035), but not the low-cash group (std. *β* = 0.04, SE 0.05, *P* = 0.418), less advanced biological age was associated with better mother-reported overall child health.

### The impact of the high-cash gift on mothers’ epigenetic indices

We found no evidence to suggest that mothers in the high-cash gift group differed from those in the low-cash gift group with respect to epigenetic measures (all effect size *d* values <0.10; Table 4 and Supplementary Table 4). These findings were robust to several sensitivity checks, including the use of non-response weights.

**Table 4:** Summary of the estimated impacts of the high-cash gift from ITT models assessing differences in maternal epigenetic indices of biological ageing and cognition in high- and low-cash transfer groups (*N* = 777)

	(1)	(2)	(3)	(4)	(5)	(6)
	Low-cash gift group mean	High-cash gift group mean	OLS with site fixed effects	OLS with baseline covariates	Effect size (*d*)	Two-tailed *P* value
DunedinPACE	−0.001	−0.000	0.001 (0.006) [−0.01 to 0.01]	0.000 (0.006) [−0.01 to 0.01]	0.00	0.981
Epigenetic-*g*_separate	0.001	−0.002	−0.004 (0.009) [−0.02 to 0.01]	−0.000 (0.009) [−0.02 to 0.02]	0.00	0.978
Epigenetic-*g*_across	0.002	−0.002	−0.003 (0.008) [−0.02 to 0.01]	0.001 (0.008) [−0.02 to 0.02]	0.01	0.925
GrimAge Accel	−0.029	0.040	0.068 (0.087) [−0.10 to 0.24]	0.105 (0.089) [−0.07 to 0.28]	0.09	0.241
PhenoAge Accel	0.060	−0.081	−0.134 (0.201) [−0.53 to 0.26]	−0.125 (0.211) [−0.54 to 0.29]	−0.05	0.552
Adjusts for data collection site			X	X	X	X
Adjusts for baseline covariates				X	X	X

Robust standard errors in parentheses, 95% CIs in brackets. Epigenetic indices were residualized for technical factors before analyses (cell type composition, slide and array). Column 3 includes fixed effects for the data collection site. Column 4 introduces covariates for child age (in months) at the age-4 visit, fixed effects for the interviewer that conducted the data collection and the following characteristics from the baseline survey: maternal characteristics (age, education, race and ethnicity, marital status, general health, depressive symptoms and cigarette and alcohol consumption during pregnancy), household characteristics (number of children born to mother, number of adults in the household, father living with the mother, household income and household net worth) and infant birth characteristics (weight at birth and gestational age). Additional covariates include child age (in months) at the age-4 visit and fixed effects for the interviewer who conducted the data collection.

### Associations among mothers’ epigenetic indices and mothers’ BMI and cognition

In maternal DNAm, Epigenetic-*g_*separate and Epigenetic-*g_*across were nearly perfectly correlated (*r* = 0.98, 95% CI 0.96 to 0.99; Table 2). Neither of the Epigenetic-*g* versions was correlated with DunedinPACE. Supplementary Table 8 reports a correlation matrix of maternal epigenetic indices and baseline covariates.

We conducted non-preregistered analyses to examine how mother’s epigenetic indices were associated with concurrent measures of BMI and executive function across both the high-cash and low-cash gift groups. As in previous studies, faster DunedinPACE was associated with higher maternal BMI (std. *β* = 0.26, 95% CI 0.18 to 0.34, SE 0.04, *P* < 0.001). Faster DunedinPACE was also associated with lower maternal executive function (std. *β* = −0.08, 95% CI −0.15 to 0.00, SE 0.04, *P* = 0.040). There was no evidence that Epigenetic-*g* was associated with maternal BMI (Epigenetic-*g*_separate: std. *β* = −0.02, 95% CI −0.10 to 0.05, SE 0.04, *P* = 0.544; Epigenetic-*g*_across: std. *β* = −0.01, 95% CI −0.08 to 0.07, SE 0.04, *P* = 0.855) or maternal executive function (Epigenetic-*g_*separate: std. *β* = 0.04, 95% CI −0.03 to 0.11, SE 0.04, *P* = 0.296*;* Epigenetic-*g_*across: std. *β* = 0.04, 95% CI −0.03 to 0.11, SE 0.04, *P* = 0.223).

## Discussion

In a randomized controlled trial in which US mothers with low income were randomized to receive unconditional cash gifts of US$333 per month or US$20 per month beginning at the time of their child’s birth, we find slower DunedinPACE of ageing among 4-year-old children in families receiving the high-cash gift. We found no evidence to suggest that children’s DunedinPACE was associated with concurrent brain activity, executive functioning, receptive vocabulary, BMI or maternal reports of overall health. In addition, we found no evidence to support our hypothesis that the high-cash gift increased children’s Epigenetic-*g*, a DNAm biomarker of cognitive function. We found inconsistent evidence that higher cash gifts were associated with lower Epigenetic-*g*. The standardized effect was similar to that for pace of ageing, but significance varied across sensitivity checks. Lower Epigenetic-*g* was weakly associated with faster epigenetic pace of ageing and more high-frequency brain activity among BFY focal children. We found no evidence of impacts on mothers’ epigenetic pace of ageing or Epigenetic-*g*. In mothers, faster pace of ageing, but not Epigenetic-*g*, was significantly associated with higher BMI and lower executive function.

In line with our expectations, this study shows that an economic intervention can slow saliva DunedinPACE, an epigenetic measure of the pace of ageing, in early childhood. In adults, DunedinPACE has been validated as a prospective predictor of ageing-related disease and mortality, and randomized trials indicate that adults’ blood-derived DunedinPACE can be modified by health-promoting interventions, such as a 25% calorie restriction over 2 years (*d* = −0.29, 95% CI −0.45 to −0.13, by the 12-month follow-up)^37^. Our findings therefore add to evidence that DunedinPACE is a promising early indicator for studying the social and behavioural antecedents of health in young humans^35–37^, raising the possibility that later BFY waves may detect downstream behavioural and health effects. Whether the modest reduction observed here (*d* = 0.17, 95% CI −0.32 to −0.01) translates into measurable health benefits remains uncertain. Because accelerated ageing accrues cumulative biological costs, economic and health returns may be especially pronounced when cellular ageing is decelerated early in life^22,40^, and even modest reductions may yield outsized public health benefits^47^. However, such benefits depend critically on the long-term persistence of this effect, which is currently unknown.

The results for epigenetic pace of ageing are especially notable given that earlier research on BFY children found no treatment effects on BMI, maternal reports of child health or development in the first three years of life, or age-4 assessments of executive function or vocabulary^15,18,20^. Developmental theories have posited that early environments shape later-life health and cognition through epigenetic canalization, even when concurrent behavioural differences are not observed^6,48–50^, yet empirical evidence in humans has been sparse. Our findings are consistent with the hypothesis that early economic conditions can influence the developing child’s epigenome even in the absence of concurrent behavioural differences.

Future work would benefit from comparing the magnitude of effects across developmental windows and intervention types. In line with the developmental origins of health and disease framework, prenatal interventions may be at least as influential as those in early childhood for shaping the epigenome. Quasi-experimental evidence indicates that prenatal exposure to famine or economic hardship can accelerate epigenetic ageing measured decades later^51,52^, motivating evaluation of prenatal cash transfers. To situate effects within the broader policy landscape, effect sizes could be benchmarked against other health-policy interventions. For instance, doubling the cumulative conditional cash transfer in Mexico from 7,500 to 15,000 pesos (US$800 to US$1,600) for children (*n* = 2,449) aged 24–68 months who had been enrolled in the programme their entire lives yielded an effect size of *d* = 0.20 (95% CI 0.09 to 0.30) on child height-for-age and *d* = −0.08 (95% CI −0.13 to −0.03) on the prevalence of childhood overweight^53^. Beyond financial support, maternal smoking during pregnancy has robust and lasting effects on the epigenome^54^. Interventions that support the family environment more broadly, recognizing that maternal health behaviours and parenting capacity are shaped by the resources and conditions available to parents, offer other plausible levers for modifying children’s epigenomes^55^. In our study, however, baseline differences in prenatal smoking or drinking were not significantly related to epigenetic indices. Instead, the effect of the cash transfer on DunedinPACE exceeded these baseline behavioural effects, highlighting cash transfers as a promising policy lever for reducing disparities in biological ageing.

Contrary to our hypothesis, we found no evidence that high-cash gifts increased children’s Epigenetic-*g*. Instead, estimates were in the unanticipated direction, although statistical significance varied across sensitivity analyses. We do not draw strong conclusions based on this finding, given the inconsistency of statistical significance across sensitivity checks and the fact that it was unanticipated. Further complicating the interpretation of Epigenetic-*g*, lower Epigenetic-*g* was weakly correlated with higher beta- and gamma-band activity at age 4. In an earlier wave of BFY data collection, 12-month-old-infants in the high-cash gift group showed some evidence of higher power in these same bands (beta and gamma, and additionally alpha). By age 4, however, only alpha power remained higher in the high-cash gift group^18^. The meaning of beta and gamma at this age remains unsettled: these bands have been linked to better cognition in some studies^56,57^ but linked to risk for language problems in other studies^58^. In the BFY sample, higher beta and gamma activity were associated with worse attention at age 4 (ref. ^18^). Overall, associations among brain activity, epigenetic measures and behaviour in this low-income BFY cohort appear to diverge from expectations based on prior observational studies of more socioeconomically diverse samples^22,44^. It is possible that, despite the lack of evidence of effects of higher-cash gifts on concurrent measures of executive functioning, receptive vocabulary, as well as behaviour and attention problems in children^15^, the cash gifts will be associated with worse cognitive outcomes later in development. Continued follow-up is needed to clarify how Epigenetic-*g* relates to longitudinal trajectories of brain development, cognition and behaviour.

The lack of evidence of effects on maternal epigenetic indices is consistent with previous BFY studies finding no evidence that the higher-cash treatment affected key environmental and behavioural factors that are consistently associated with adult biological ageing. Specifically, although the higher-cash gifts increased net household income^14^, there is no evidence that they improved housing quality, encouraged moves to better neighbourhoods or reduced economic hardship or food insecurity^14^. Other studies similarly found that there was no detectable effect of cash gifts on many environmental and behavioural factors known to affect mothers’ mental and physical health^14,16,59,60^.

We acknowledge several limitations to this work. First, the BFY mothers and children are still young, so we do not know if and how epigenetic modifications in response to the cash transfer may emerge, persist and relate to ageing, health and cognitive outcomes. Because the cash gifts ended shortly after the focal child’s sixth birthday, it is also unclear whether any differences will endure without continued economic support. We also lack epigenetic measurements before age 4, so we do not know exactly when cash transfer impacts on children’s epigenome first emerged. Moreover, this study does not include other maternal physical health and ageing biomarkers (for example, multimorbidity counts and blood-chemistry biomarkers) that may be more sensitive to cash transfer effects.

Second, researchers disagree about how to best interpret epigenetic indices of biological ageing and cognition that are generated in adults and then applied to young children, a debate situated in a larger theoretical disagreement about what, exactly, epigenetic biological ageing is and how it is related to child development^33^. Our finding that the high-cash gift modified the epigenetic pace of biological ageing in children offers support for the notion that epigenetic development and ageing are, to some degree, connected and modifiable^22,25^.

Third, the epigenetic biomarkers examined here were developed in adult blood DNAm and then applied to saliva DNAm, which is easier to collect than blood. DNAm differs between tissue types, so we corrected for cell composition differences between individuals. Both blood and saliva are rich in immune cells, which are especially important to biological ageing and may explain the consistent associations with social determinants of health in studies leveraging saliva^35,61^. While some signal is lost in cross-tissue applications and may attenuate our reported effects^62^, our research demonstrates that saliva is a promising avenue for measuring the human epigenome and, thus, deserves attention as a peripheral substrate for biomarker development.

Fourth, the epigenetic measures we generated were developed in cohorts of predominantly white European participants. Although analyses of race and ethnic variation in epigenetic clocks may be confounded by ancestry-linked genetic artefacts^63,64^, there is currently little evidence of such bias for DunedinPACE and Epigenetic-*g*; studies report consistent associations with health and cognition within and across different racial and ethnic groups^31,42,65–69^. Nevertheless, an urgent priority is to develop and validate inclusive, globally representative epigenetic clocks with careful calibration for ancestry, age range, array platform and tissue type.

In conclusion, providing mothers with low income a monthly unconditional cash transfer of US$333 per month during the first four years of their child’s life yielded no evidence of impact on maternal epigenetic indices, but slowed children’s epigenetic pace of biological ageing. Effects on children’s Epigenetic-*g* were inconclusive. These results suggest that antipoverty interventions may decelerate epigenetic ageing in early childhood, when cellular ageing processes are proposed to originate and may be most responsive to environmental interventions^34,70^. Because adults with slower epigenetic ageing tend to live longer and healthier lives^28,71,72^, our findings suggest that reducing early childhood poverty through direct cash transfers may have favourable impacts on recipients’ health. Future research is needed to understand whether these epigenomic effects persist and translate into future favourable developmental and health outcomes.

## Methods

This research complies with all relevant ethical regulations. All study procedures were approved by the institutional review boards of Teachers College, Columbia University (protocol #18-210), and the New York State Psychiatric Institute (protocol #7606). Informed consent was obtained from all participating mothers.

BFY is a large, multiwave randomized controlled trial with numerous preregistered outcomes reported across several companion publications. The trial’s primary preregistered outcomes—measures of children’s cognitive, behavioural and brain development, used to determine the trial’s overall sample size—are reported in a paper combining findings from the age-4 and age-6 waves (Magnuson et al., under review). Secondary and exploratory outcomes from the age-4 wave are reported in refs. ^14,17,20,59,73–76^. DunedinPACE-pace of aging and Epigenetic-*g* were designated as secondary outcomes of the BFY trial before age-4 data collection (clinical trial identifier NCT03593356), together with the supplemental biological age acceleration measures examined here (PhenoAge Acceleration and GrimAge Acceleration). After data collection concluded but before generating epigenetic data, we publicly preregistered a detailed analysis plan for the present study (https://osf.io/8grhj). We confirm that we are reporting the hypotheses, predictions, outcome variables and analyses exactly as preregistered, except if otherwise indicated. All secondary outcomes related to the epigenetic research questions addressed in this Article are reported here.

Because study design, recruitment, randomization, the cash-gift intervention and several measures are shared across BFY companion papers, portions of Methods below—not limited to the description of measures—are reproduced from our earlier publications reporting on this trial^13–17,19,20,76^. The authors of this Article overlap substantially with the authorship of each of these companion papers. Where the same passages also appear in earlier companion papers, we have paraphrased the text below.

### Participants

The US-based study BFY uses a randomized controlled trial design to estimate effects of early childhood cash transfers^13^. The full 1,000-dyad sample was enrolled between 9 May 2018, and 19 June 2019^13,14^. The study recruited mothers and their newborns across 12 birth hospitals in four areas: the greater New Orleans metropolitan area, the greater Omaha metropolitan area, the Twin Cities (Minneapolis and St. Paul) and New York City. Following childbirth, mothers were randomly allocated into two study arms: a high-cash cohort (US$333 per month, *n* = 400) or a low-cash cohort (US$20 per month, *n* = 600) to receive unconditional monthly cash transfers. Originally scheduled for the first 40 months of the child’s life, the intervention was later lengthened to the first 76 months. The final payment was distributed on 18 September 2025. Consequently, both enrolment and the intervention have concluded, while cohort data collection and research evaluation remain ongoing.

Providing this cash support resulted in a yearly boost of roughly US$4,000, representing an approximate 18% growth in overall family income. To be eligible, a mother’s self-reported income in the prior calendar year had to fall below the US federal poverty threshold for their family size. Additional inclusion criteria required that the mother was of legal age for informed consent; spoke English or Spanish; resided in the state of recruitment; and indicated she was unlikely to move out of the state or country within the next 12 months. Furthermore, the infant had to be a singleton pregnancy admitted to the newborn nursery (not intensive care) and discharged into the custody of the mother.

The randomized allocation sequence was generated separately for each of the four study sites. Staff built a 250-row roster per site, comprising 150 rows reserved for the low-cash assignment and 100 for the high-cash assignment. These sequences were randomly shuffled and maintained on a secure server at the University of Michigan Survey Research Center. Because recruitment fell short of the 250-participant target in the Twin Cities, additional allocation rows were generated for the remaining three sites using an identical procedure, ensuring the aggregate sample preserved the intended 40%:60% high- to low-cash allocation. Randomization successfully achieved baseline equivalence across 27 characteristics for the full enrolled sample within each site^13^.

Baseline interviews were administered by trained interviewers using Blaise computer-assisted personal interviewing software (version 4.8). Upon interview completion and agreement to participate, the software connected to a study-specific web application to verify site and respondent identifiers. The system then sequentially drew the next unclaimed ID from that site’s randomized roster, fulfilling requests in the order received regardless of the recruitment hospital. Once the assigned monthly amount was displayed for interviewer confirmation, the interviewer disclosed the payment tier to the participant and activated a ‘4MyBaby’ debit card at the hospital. Ongoing monthly cash gifts were loaded onto this card on the evening before the child’s monthly birth anniversary (see ref. ^77^ for details about the cash gift). Efforts were made to ensure that, to the greatest possible extent, the cash gift did not affect the mother’s eligibility for safety net programmes.

Sample size for the BFY trial was determined a priori based on its primary developmental outcomes. Assuming approximately 20% attrition by the trial’s capstone data-collection wave, the anticipated analytic sample of 800 mother–child dyads (40%:60% high-cash:low-cash allocation) was calculated to provide 80% power to detect a standardized effect size of *d* = 0.207 at a two-tailed alpha of 0.05. A separate a priori power calculation specific to the epigenetic secondary outcomes reported here was not conducted. Retention to date has been high, with over 89% (*n* = 890) of mothers participating at the age-1 through age-4 waves of follow-up and 78% of mothers and 74% of children providing epigenetic data. Analyses used all mother–child dyads with quality-controlled DNAm data available at the age-4 visit (children *n* = 735; mothers *n* = 777), a sample of a similar order of magnitude to that anticipated in the trial’s original power calculation.

Mothers self-identified as approximately 40% Black, 40% Latinx and 20% ‘other’ or ‘white’ or ‘Asian’.

Table 5 reports descriptive statistics.

**Table 5:** Descriptive statistics

	Mean	s.d.	Minimum	Maximum
Child variables				
Chronological age (years)	4.02	0.18	3.83	4.92
DunedinPACE	1.48	0.09	1.00	1.77
Epigenetic-*g*_separate^a^	0.00	0.22	−0.60	1.15
Epigenetic-*g*_across^a^	−0.18	0.19	−0.72	0.61
PhenoAge^b^	46.86	8.74	16.51	68.38
GrimAge^b^	41.70	2.81	32.44	48.37
Maternal variables				
Chronological age (years)	31.66	5.91	22.00	52.42
DunedinPACE	1.56	0.10	1.10	1.90
Epigenetic-*g*_separate^a^	0.00	0.20	−0.60	0.85
Epigenetic-*g*_across^a^	0.17	0.18	−0.27	0.97
PhenoAge^b^	76.84	10.04	49.52	116.65
GrimAge^b^	62.26	5.00	48.02	81.78

Sample restricted to those with a valid epigenetic sample at the age-4 timepoint. None of the described epigenetic scores was residualized for technical factors or chronological age or standardized. By contrast, epigenetic scores used in the analyses were residualized for technical factors and chronological age, and then standardized separately for maternal (*N* = 777, mean 0, s.d. 1) and child DNAm samples (*N* = 735, mean 0, s.d. 1). ^a^One robustness check differentiated Epigenetic-*g* computed separately for maternal and child DNAm (that is, Epigenetic-*g*_separate) versus Epigenetic-*g* computed across maternal and child DNAm together (that is, Epigenetic-*g*_across). ^b^A mean increase is a common observation in both saliva and buccal epigenetic clock applications when those were originally developed in blood^61^.

### DNAm procedure

During the age-4 university visit (which occurred July 2022 to August 2023), all mothers were invited to provide saliva samples from themselves and their children for DNAm analysis as an optional study extension. Informed consent was obtained after participants viewed an information video. Mother and child saliva samples (2 ml) were collected using ORAcollect•Dx (OCD-100/100A, DNA Genotek). Saliva samples were stored in Oragene kits that release a DNA stabilizing buffer at room temperature until shipment to the Max Planck Institute of Psychiatry in Germany. The researchers who collected the saliva samples were blind to group assignment.

The *n* = 1536 samples from mothers and children (including technical replicates) were randomized by sex and collection site across 16 plates, each with 96 wells, using the R package ‘Omixer’^78,79^. Family identifiers were used as blocking variables to ensure all mother-child pairs were grouped on the same column of a plate. Each column of the 96-well plates consisted of approximately four treatment and four control samples, which were randomly ordered. We selected 18 samples (9 mothers and 9 children) with concentrations higher than 200 ng µl^−1^ as technical replicates to probe methylation profiling reliability. *N* = 8 samples were excluded on the basis of DNAm quality control, of which two were technical replicates. The resulting final sample of *n* = 1512 included 729 complete mother–child pairs and 54 incomplete pairs (48 mother-only samples and 6 child-only samples). There were *n* = 735 children (*n* = 369 boys and *n* = 366 girls), of which *n* = 312 were in the high-cash and *n* = 423 in the low-cash group. There were *n* = 777 mothers, of which *n* = 329 were in the high-cash and *n* = 448 in the low-cash group. On a CpG probe level, 885,195 probes passed quality control criteria.

### DNAm preprocessing

DNA extraction and methylation processing using the Infinium MethylationEPIC v2.0 Kit (Illumina) to assess methylation levels at 936,990 methylation sites is currently being conducted by the Max Planck Institute of Psychiatry, Department Genes and Environment, Munich, Germany.

Quality control and normalization of the methylation data were conducted by the Max Planck Institute of Human Development, MPRG Biosocial, Berlin, Germany, using R software (R Core Team, 2021). The pipeline, which can be found at https://github.com/Biosocial/BFY-and-Epigenetic-Aging-, was largely in line with the preprocessing steps performed in previous cohorts (for example, Texas Twin Project, Future Families and Child Well-Being, Netherland Twin Registry; personal communication). We used the ‘minfi’ package for most preprocessing steps^80^. Within-array normalization will be applied to address issues related to array background correction, red/green dye bias and probe type I/II correction. The Noob preprocessing method, implemented as ‘preprocessNoob’^81^, performs background correction and dye-bias equalization, producing similar normalization effects on the data as probe type correction methods such as BMIQ^82,83^.

CpG probes were excluded if they (1) had a detection of *P* > 0.01, (2) had fewer than four beads in more than 5% of the samples or (3) had a success rate <0.95 across all samples^84^. Probes on the X or Y chromosome will not be excluded for the analyses^85^. Samples were excluded if they (1) showed low intensity probes as indicated by the log of average methylation <9 and (2) their detection *P* is >0.01 in >10% of their probes.

Cell composition of five cell types were estimated using reference-free cell composition, using the ‘RefFreeEWAS’ R package^86^. We have found cell composition correction using this reference-free estimation to result in the highest blood–saliva cross-tissue correlation point estimates compared with reference-based methods^62^.

In line with previous cohorts, such as the Future Families and Child Well-Being study, we identified and corrected sample swaps for 11 mother–child dyads. These corrections were based on discrepancies between chronological age and DNAm age estimates derived from the Horvath skin and blood clock, which can quantify chronological age in saliva with very high precision (*R*^2 ^> 90%)^87^. All mismatched ages were part of a mismatched dyad, indicating that the samples labelled as children were actually from mothers, and vice versa.

### Measures

#### Epigenetic outcomes

DunedinPACE-pace of aging was developed in the Dunedin Study birth cohort and is based on analyses of within-person change in over 19 years of follow-up in 19 system-integrity biomarkers measured repeatedly at age 26, 32, 38 and 45 (ref. ^88^). Increments of methylation pace of ageing correspond to ‘years’ of physiological change occurring per 12 months of chronological time. Values >1 indicate accelerated ageing. Values <1 indicate slowed ageing. DunedinPACE was residualized for array, slide, cell composition (top five principal components (PCs) from reference-free cell estimation) and then standardized (mean 0, s.d. 1). DunedinPACE-pace of aging is a preregistered, secondary outcome.

Epigenetic-*g* is modelled on the basis of general cognitive functioning (*g*) in adults from the Generation Scotland Study ages 18 to 93, controlling for their chronological age^41^. General cognitive ability was derived from the first unrotated principal component of logical memory, verbal fluency and digit symbol tests, and vocabulary. A higher score indicates a DNAm profile that more closely resembles the methylation profile of adults from the Generation Scotland Study who performed higher on tests of general cognitive function.

One of our robustness checks differentiated Epigenetic-*g* computed separately for maternal and child DNAm (that is, Epigenetic-*g*_separate) versus Epigenetic-*g* computed across maternal and child DNAm together (that is, Epigenetic-*g*_across). Unlike epigenetic clocks, Epigenetic-*g* includes a scaling function that normalizes DNAm at individual DNAm sites across people within the sample, which may account for technical factors in DNAm batches but could also introduce age-related variation in samples of a wide age range. Because the original algorithm and our preregistered analysis plan lacked specificity about whether to compute Epigenetic-*g* separately or across maternal and child DNAm samples, we considered this an important robustness check.

For both iterations of Epigenetic-*g*, Epigenetic*-g* was—separately for maternal and child DNAm samples—residualized for array, slide and cell composition (top five PCs from reference-free cell estimation) and then standardized (mean 0, s.d. 1), in line with our preregistration. Epigenetic-*g* is a preregistered, secondary outcome.

GrimAge is a DNAm measure developed on a set of physiological indicators using machine learning analyses and DNAm algorithms to predict morbidity and mortality^89^. A person’s GrimAge signifies the age in years at which average mortality risk in the Framingham Heart Study Offspring cohort matches the person’s epigenetically-predicted mortality risk. We used DNAm principal components when computing GrimAge to increase reliability^90^, and created GrimAge Acceleration by residualizing GrimAge for chronological age. GrimAge was residualized for array, slide and cell composition (top five PCs from reference-free cell estimation) and then standardized (mean 0, s.d. 1). GrimAge Acceleration is a preregistered, supplemental epigenetic measure of biological ageing (see ‘Statistical analyses’ section (a)).

PhenoAge was modelled on the basis of physiological markers and chronological age and subsequently applied to a new sample to derive a final DNAm clock^45^. It represents the age in years at which average mortality risk in the Third National Health and Nutrition Examination Survey (NHANES III) matches the mortality risk predicted by the PhenoAge algorithm. We used DNAm principal components when computing PhenoAge to increase reliability^90^ and created PhenoAge Acceleration by residualizing PhenoAge for chronological age. PhenoAge was residualized for array, slide and cell composition (top five PCs from reference-free cell estimation) and then standardized (mean 0, s.d. 1). PhenoAge Acceleration is a preregistered, supplemental epigenetic measure of biological ageing (see ‘Statistical analyses’ section (a)).

Additional measures are partly reproduced from earlier publications.

#### Health

Child health was assessed using maternal ratings on a 5-point Likert scale ranging from 1 (excellent) to 5 (poor). In addition, age-4 BMI was calculated from laboratory-measured height and weight, then converted into sex- and age-specific *z* scores using standard US Centers for Disease Control and Prevention guidelines (https://www.cdc.gov/growthcharts/percentile_data_files.htm).

#### Nutrition

We deviated from the preregistration and did not include this measure as it was not available at age 4 years.

#### Language and cognition

At age 4, children’s executive functions were evaluated in a laboratory setting using the Minnesota Executive Function Scale^91^. Concurrently, early literacy and receptive vocabulary skills were determined via the Receptive One-Word Picture Vocabulary Test^92^, with monolingual and bilingual assessments analysed independently.

#### Brain activity in EEG measurements

To evaluate resting neural function at age 4, EEG data using 128-channel Geodesic Sensor Nets (Magstim EGI) were collected (see ref. ^93^ for age-4 impacts). Before field implementation, the research team validated the deployment strategy through pilot testing and focus groups^19,94^. During the 3-min recording session, participants maintained a distance of 60 cm from a display showing silent, non-social toy videos while impedances were targeted below 50 kΩ. Data sampling occurred at 1,000 Hz using an online Cz reference. Blended data processing utilized EEGLAB, custom MATLAB routines and the MADE pipeline under blinded conditions. Spectral power was categorized into four primary frequencies: theta (3–6 Hz), alpha (7–12 Hz), beta (13–20 Hz) and gamma (21–45 Hz), evaluating relative power against total absolute power. In addition, regional analyses categorized electrodes into standard localized brain areas (frontal, central, parietal, temporal and occipital).

We expected Epigenetic-*g* to be positively correlated with the high-power bands and negatively correlated with the low-power bands. Relative power in these four bands was our main variable of interest. Supplementary analyses also examined regional power grouped into central (6, 112, 105, 87, 79, 54, 37, 30, 13, 106, 80, 55, 31, 7, Cz), frontal (20, 12, 5, 118, 24, 19, 11, 4, 124, 27, 23,18, 16, 10, 3, 123), temporal (40, 46, 39, 45, 50,109, 102, 115, 108, 101), parietal (53, 61, 62, 78, 86, 52, 60, 67, 72, 77, 85, 92) and occipital (66, 71, 76, 84, 70, 75, 83) regions.

#### CBCL total behaviour problems

Behavioural problems were evaluated using the CBCL^95^. Caregivers filled out four specific subscales: anxiety/depression, aggressive behaviour, attention problems and emotionally reactive. A comprehensive score was then derived by adding up the raw values from each subscale. This was not a preregistered outcome, but added for completeness.

#### PSRA attention/impulse control

Once the age-4 laboratory appointment was finished, the primary research assistant completed the PSRA^96^. The attention/impulse control subscale was examined in the present study. This was not a preregistered outcome, but added for completeness.

### Statistical analyses

(a) Analyses examining the impact of the high-cash gift among mothers with low income and their children on epigenetic indices of pace of ageing and cognition.

We examined intervention effects on epigenetic indices preregistered as clinical trial outcomes prior to the onset of age-4 data collection for both children and mothers: DunedinPACE-pace of aging^88^ and Epigenetic-*g*^41^. We supplemented these analysis by reporting group differences in two proposed metrics of biological age acceleration—the PhenoAge Acceleration^45^ and GrimAge Acceleration^46^—which we did not expect to be sensitive to financial circumstances children experience based on previous studies, but are worth reporting, because they are among the strongest predictors of all-cause morbidity, mortality and physiological decline in adults^97^. We grouped analysis of PhenoAge and GrimAge Acceleration in the same family of tests and planned to adjust for multiple testing using false discovery rate (FDR) correction, because these DNAm measures are both tapping into the construct of ‘accelerated biological ageing’^98^.

Because assignment to the high-cash gift group was random by design, securing an unbiased estimate of the impact of the higher cash gift (a difference of US$313 per month between groups) is relatively straightforward. Our estimates were derived by ITT estimates from the following model included in Code S3, which can be found at the Inter-university Consortium for Political and Social Research (OPEN ICPSR; ID159422; https://www.openicpsr.org/openicpsr/project/159422/version/V8/view): $$Y=\,Z{\rm{\pi }}+{\rm{X}}{{\beta }}+{{\varepsilon }}$$ where *Z* is a dummy-coded indicator of whether a mother is in the high-cash group (1 = high-cash gift group, 0 = low-cash gift group), and thus *π* is the causal estimate of receipt of the high-cash gift. A 1-unit difference in *Z* corresponds to the difference between control and treatment. Treatment is the higher value so that a positive value of *π* is a positive treatment effect. *Y* is the outcome of interest for child or mother, and *ε* is the error term. The model will include a vector of ones for the intercept and pretreatment baseline covariates (*X*, see above). Given the implementation success of the debit card mechanism, the ITT estimate captures the effects of a net positive income shock of US$313 per month (which is the absolute difference between high- and low-cash receiving groups), essentially equivalent to a treatment-on-the-treated interpretation. ITT estimates were converted to effect sizes.

Analyses included the following preregistered pretreatment baseline covariates: mother’s characteristics (age, education, race and ethnicity, marital status, health, depressive symptoms, and cigarette and alcohol consumption during pregnancy), household characteristics (number of children born to mother, number of adults in the household, father living with the mother, household income and household net worth) and infant’s birth characteristics (sex, weight at birth and gestational age). Note that, in the context of a randomized experiment, the intention in using covariates is to increase power by reducing unexplained variance, and thus reduce the standard errors of the average treatment effect estimate, and to help to adjust for baseline imbalances in the analysis samples (whose expected values on pretreatment variables are equivalent). Epigenetic indices were residualized for technical factors in a prior step (plate, assay and cell composition; see ‘Measures’ section). We added a covariate for the interviewer who conducted the age-4 assessment to account for any differences across assessors.

(b) Analyses examining whether there is an interaction between treatment group and epigenetic indices in the prediction of BMI, health, nutrition, language and cognition, and neural activity.

These analyses included in Code S4, which can be found at the Inter-university Consortium for Political and Social Research (OPEN ICPSR; ID159422; https://www.openicpsr.org/openicpsr/project/159422/version/V8/view), were treated as exploratory with no directional hypothesis tested.

First, we examined how epigenetic indices were correlated with child developmental outcomes across the whole sample. We regressed child developmental outcomes on DunedinPACE-pace of aging, separately for each measure of BMI, health, nutrition, language and cognition, and brain activity listed in the ‘Measures’ section. We regressed child developmental outcomes on Epigenetic-*g*, separately for each measure of language, cognition and brain activity. For both DunedinPACE and Epigenetic-*g* models, we included child’s age in months and sex as a covariate, given previous reports of age and sex differences in epigenetic indices^22^. Models examining brain activity also controlled for the final number of usable EEG epochs.

Second, we tested whether the cash gift treatment moderated associations between epigenetic indices and early child developmental outcomes (BMI, health, nutrition, language and cognition, and neural activity). This is mathematically equivalent to testing whether the cash gift treatment has heterogeneous treatment effects on child developmental outcomes depending on epigenetic indices. We derived ITT estimates that regress child developmental outcomes with high- versus low- cash gift receiving group, epigenetic indices and a group-by-epigenetic-indices interaction.

$$Y={\beta }_{0}+{\pi }_{0}Z+{\beta }_{1}\times \mathrm{Epi}+i\times \left(Z\times \mathrm{Epi}\right)+\sum {\beta }_{k}\times {X}_{k}+{\varepsilon },$$

where ${{\rm{\pi }}}_{0}$ is the average treatment effect at epigenetic indices Epi = 0 (centred, sample mean), ${\beta }_{1}$ is the average effect of Epi when group = 0 (that is, dummy-coded control group), *i* is the treatment-by-Epi interaction effect and ${\beta }_{k}$ is the effect of covariate *k*. We controlled for covariate-by-treatment interactions to assess their impact on the main effects not shown^99^. After confirming main effects of interest were not affected, we removed these interactions afterwards owing to variance inflation concerns.

This interaction model can be rewritten (covariate effects not shown) as

$$Y={\beta }_{0}+{{{\pi }}}_{0}Z+\left({\beta }_{1}+i \times Z\right){\rm{Epi}}+{{\varepsilon }}$$

to highlight that the coefficient Epi is itself dependent on the treatment, or as

$$Y={\beta }_{0}+\left({\pi }_{0}+i\times \mathrm{Epi}\right)Z+{\beta }_{1}\times \mathrm{Epi}+\varepsilon$$

to highlight that the coefficient on the treatment is itself dependent on Epi.

Where a significant interaction was detected, we conducted simple slopes analysis at different levels of the moderator (for example, high- versus low-cash-receiving group; fast versus slow pace of ageing) to inform whether cash gift group or epigenetic indices are acting as the moderator.

We augmented our analyses by reporting associations with two proposed metrics of biological age acceleration, the PhenoAge Acceleration and GrimAge Acceleration, because they are among the strongest predictors of all-cause morbidity, mortality and physiological decline in adults^97^. We will treat these epigenetic indices as supplementary to our main epigenetic indices. We will group analyses PhenoAge and GrimAge Acceleration in the same family of tests and adjust for multiple testing using FDR correction, because these measures are both tapping into the construct of ‘accelerated biological ageing’ and will be combined in one final conclusion^98^.

### Reporting summary

Further information on research design is available in the Nature Portfolio Reporting Summary linked to this article.

## Supplementary information

Supplementary Information Supplementary Tables 1–8.

Reporting Summary

## Data Availability

Summarized data, except for child chronological age due to HIPAA data-sharing restrictions, and the corresponding documentation are publicly available at the Inter-university Consortium for Political and Social Research (OPEN ICPSR; ID159422; https://www.openicpsr.org/openicpsr/project/159422/version/V8/view).

## Code availability

Code for DNA methylation preprocessing and epigenetic score computation is available via GitHub at https://github.com/Biosocial/BFY-and-Epigenetic-Aging as well as at the Inter-university Consortium for Political and Social Research (OPEN ICPSR; ID159422; https://www.openicpsr.org/openicpsr/project/159422/version/V8/view).
