# Supplementary material for: Effects of a randomized controlled trial of unconditional cash transfers on epigenetic measures of ageing and cognition in children and mothers
Source: Nat Hum Behav. 2026 Sep 8;10(10):2002–15. doi: 10.1038/s41562-026-02568-4 (PMC13683044; doi:10.1038/s41562-026-02568-4)
Supplement: Supplementary file 1 [file 41562_2026_2568_MOESM1_ESM.pdf]

nature human behaviour

Supplementary information

https://doi.org/10.1038/s41562-026-02568-4

Effects of a randomized controlled trial of unconditional cash transfers on epigenetic measures of ageing and cognition in children and mothers

In the format provided by the authors and unedited

Supplementary Results

Table of contents

Table S1.

Sample Characteristics and Baseline Balance for the Low- and High-Cash Gift Groups in the Epigenetic Analytic Sample (n = 783).

Table S2.

Continued from Table S1. Sample Characteristics and Baseline Balance for the Low- and High-Cash Gift Groups in the Epigenetic Analytic Sample (n = 783).

Table S3.

Correlation matrix of child epigenetic indices and baseline covariates.

Table S4.

Weighted impacts of the cash gift on maternal and child epigenetic profiles in sensitivity analysis weighting for non-response.

Table S5.

Standardized parameter estimates from a model where child EEG regional absolute power measures were regressed on epigenetic indices.

Table S6. Interaction of the cash gift and child epigenetic profiles on health, cognition, and relative EEG power.

Table S7.

Interaction of the cash gift and child epigenetic profiles on health, cognition, and relative EEG power.

Table S8. Correlation matrix of maternal epigenetic indices and baseline covariates.

Table S1.

Sample Characteristics and Baseline Balance for the Low- and High-Cash Gift Groups in the Epigenetic Analytic Sample (n = 783).

	Total Sample		Low-Cash Gift Group		High cash gift group		Standardized Mean Difference		P-value
	Mean (SD)	N	Mean (SD)	N	Mean (SD)	N	Hedges' g	Cox's Index	
Child is female	0.498	783	0.507	450	0.486	333		-0.051	0.57
Child weight at birth (pounds)	7.121	781	7.152	449	7.080	332	-0.070		0.32
	(1.020)		(1.033)		(1.002)				
Child gestational age (weeks)	39.073	779	39.112	446	39.020	333	-0.076		0.34
	(1.217)		(1.192)		(1.249)				
Mother age at birth (years)	27.184	783	26.951	450	27.498	333	0.092		0.17
	(5.920)		(5.969)		(5.848)				
Mother's education, years	11.910	775	11.919	444	11.897	331	-0.008		0.97
	(2.813)		(2.672)		(2.995)				
Mother race/ethnicity: white, non-Hispanic	0.091	783	0.093	450	0.087	333		-0.044	0.61
Mother race/ethnicity: Black, non-Hispanic	0.398	783	0.382	450	0.420	333		0.096	0.21
Mother race/ethnicity: multiple, non-Hispanic	0.038	783	0.044	450	0.030	333		-0.241	0.27
Mother race/ethnicity: other or unknown	0.036	783	0.051	450	0.015	333		-0.764	0.00
Mother race/ethnicity: Hispanic	0.437	783	0.429	450	0.447	333		0.044	0.41
Mother marital status: never married	0.447	783	0.427	450	0.474	333		0.115	0.17
Mother marital status: single, living with partner	0.239	783	0.258	450	0.213	333		-0.152	0.16
Mother marital status: married	0.227	783	0.220	450	0.237	333		0.058	0.54

Note. Sample is restricted to families who contributed a saliva sample for epigenetic analyses (mother or child). P-values were derived from a series of OLS bivariate regressions in which each baseline characteristic was regressed on the treatment status indicator using robust standard errors and site-level fixed effects. Standardized mean differences were calculated using Hedges' g for continuous variables and Cox's Index for dichotomous variables. If there were more than 10 missing cases for a covariate, missing data dummies were included in the table and in the joint test. If there were less than 10 cases missing, missing data dummies were not included in the table but included in the joint test.

Table S2.

Continued from Table S1. Sample Characteristics and Baseline Balance for the Low- and High-Cash Gift Groups in the Epigenetic Analytic Sample (n = 783).

	Total Sample		Low-Cash Gift Group		High cash gift group		Standardized Mean Difference		P-value
	Mean (SD)	N	Mean (SD)	N	Mean (SD)	N	Hedges' g	Cox's Index	
Mother marital status: divorced/ separated	0.040	783	0.044	450	0.033	333		-0.181	0.39
Mother marital status: other or unknown	0.047	783	0.051	450	0.042	333		-0.123	0.44
Mother health is good or better	0.898	783	0.882	450	0.919	333		0.253	0.09
Mother depression (CESD)	0.686 (0.457)	783	0.690 (0.459)	450	0.679 (0.455)	333	-0.024		0.74
Cigarettes per week during pregnancy	3.850 (15.995)	777	4.375 (18.870)	445	3.147 (11.012)	332	-0.077		0.21
Alcohol drinks per week during pregnancy	0.112 (1.404)	780	0.173 (1.816)	448	0.030 (0.418)	332	-0.102		0.11
Number of children born to mother	2.425 (1.409)	783	2.362 (1.408)	450	2.511 (1.409)	333	0.105		0.17
Number of adults in household	2.079 (0.976)	783	2.113 (0.987)	450	2.033 (0.961)	333	-0.082		0.29
Biological father lives in household	0.379	783	0.402	450	0.348	333		-0.140	0.14
Household combined income	21,771.91 (17,931.67)	736	22,362.32 (18,968.04)	427	20,956.04 (16,387.33)	309	-0.078		0.32
Household income unknown	0.060	783	0.051	450	0.072	333		0.222	0.23
Household networth	-1,359.01 (22,814.47)	699	-747.14 (27,922.34)	402	-2,187.21 (13,037.39)	297	-0.063		0.37
Household net worth unknown	0.107	783	0.107	450	0.108	333		0.006	0.94
Joint Test of Orthogonality: Chi2(28)=33.16, *p*= 0.23, *n*= 783									

Note. Sample is restricted to families who contributed a saliva sample for epigenetic analyses (mother or child). P-values were derived from a series of OLS bivariate regressions in which each baseline characteristic was regressed on the treatment status indicator using robust standard errors and site-level fixed effects. The joint test of orthogonality was conducted across measures listed in Table S1 and S2 using a probit model with robust standard errors and site-level fixed effects. Standardized mean differences were calculated using Hedges' g for continuous variables and Cox's Index for dichotomous variables. If there were more than 10 missing cases for a covariate, missing data dummies were included in the table and in the joint test. If there were less than 10 cases missing, missing data dummies were not included in the table but included in the joint test.

Table S3-1. Correlation matrix of child epigenetic indices and baseline covariates.

Variables	1	2	3	4	5	6	7	8	9	10	11	12	13	14
(1) Child DunedinPACE	1													
(2) Child Epigenetic-g_across	-0.06<br>(0.09)<br>[-.13, .01]	1												
(3) Child Epigenetic-g_separate	-0.10*<br>(0.01)<br>[-.17, -.03]	0.97*<br>(0.00)<br>[.97, .97]	1											
(4) Child PhenoAge Accel.	0.11*<br>(0.00)<br>[.04, .18]	0.01<br>(0.73)<br>[-.06, .09]	0.04<br>(0.33)<br>[-.04, .11]	1										
(5) Child GrimAge Accel.	0.18*<br>(0.00)<br>[.11, .25]	-0.04<br>(0.33)<br>[-.11, .04]	-0.04<br>(0.27)<br>[-.11, .03]	0.51*<br>(0.00)<br>[.45, .56]	1									
(6) Child is female	0.01<br>(0.69)<br>[-.10, .07]	0.01<br>(0.82)<br>[-.05, .12]	0.01<br>(0.81)<br>[-.05, .12]	-0.03<br>(0.49)<br>[-.14, .03]	-0.06<br>(0.13)<br>[-.16, .00]	1								
(7) Child Birthweight	0.01<br>(0.79)<br>[-.07, .10]	-0.02<br>(0.58)<br>[-.10, .07]	-0.03<br>(0.48)<br>[-.10, .06]	0.02<br>(0.57)<br>[-.06, .10]	-0.02<br>(0.59)<br>[-.12, .04]	-0.16*<br>(0.00)<br>[-.23, -.07]	1							
(8) Child Gestational Age	0.00<br>(0.92)<br>[-.11, .06]	0.07<br>(0.07)<br>[.02, .19]	0.06<br>(0.08)<br>[.02, .18]	0.03<br>(0.49)<br>[-.07, .10]	0.01<br>(0.85)<br>[-.09, .08]	0.02<br>(0.51)<br>[-.04, .12]	0.38*<br>(0.00)<br>[.31, .45]	1						
(9) Avg cigs per week during pregnancy	-0.02<br>(0.65)<br>[-.11, .05]	-0.01<br>(0.75)<br>[-.10, .07]	-0.01<br>(0.82)<br>[-.09, .08]	-0.08*<br>(0.02)<br>[-.18, -.01]	0.00<br>(0.99)<br>[-.09, .08]	0.01<br>(0.71)<br>[-.06, .11]	-0.04<br>(0.23)<br>[-.12, .05]	-0.13*<br>(0.00)<br>[-.24, -.00]	1					
(10) Avg drinks per week during pregnancy	0.00<br>(0.90)<br>[-.09, .08]	0.04<br>(0.33)<br>[-.04, .13]	0.03<br>(0.38)<br>[-.04, .13]	-0.01<br>(0.87)<br>[-.09, .08]	0.00<br>(0.97)<br>[-.09, .08]	0.01<br>(0.83)<br>[-.08, .08]	-0.01<br>(0.87)<br>[-.07, .10]	0.00<br>(0.98)<br>[-.07, .10]	0.19*<br>(0.00)<br>[.14, .30]	1				
(11) Household Income	0.07<br>(0.07)<br>[-.01, .16]	-0.02<br>(0.60)<br>[-.12, .05]	-0.03<br>(0.49)<br>[-.12, .04]	-0.05<br>(0.17)<br>[-.14, .03]	-0.02<br>(0.65)<br>[-.09, .07]	0.01<br>(0.85)<br>[-.06, .10]	0.03<br>(0.36)<br>[-.06, .10]	0.03<br>(0.47)<br>[-0.06 .11]	-0.03<br>(0.48)<br>[-.12, .05]	0.01<br>(0.76)<br>[-.07, .09]	1			
(12) Household Net worth	0.03<br>(0.39)<br>[-.04, .12]	0.05<br>(0.24)<br>[-.03, .13]	0.03<br>(0.46)<br>[-.05, .11]	-0.01<br>(0.89)<br>[-.08, .08]	-0.03<br>(0.49)<br>[-.10, .06]	-0.02<br>(0.46)<br>[-.07, .10]	-0.06<br>(0.05)<br>[-.17, -.01]	-0.04<br>(0.21)<br>[-.10, .07]	-0.03<br>(0.34)<br>[-.11, .06]	0.00<br>(0.91)<br>[-.09, .08]	0.16*<br>(0.00)<br>[.10, .26]	1		
(13) Completed H.S.	0.03<br>(0.41)<br>[-.06, .11]	-0.05<br>(0.22)<br>[-.15, .02]	-0.05<br>(0.16)<br>[-.16, .01]	0.13*<br>(0.00)<br>[.02, .19]	0.10*<br>(0.01)<br>[.00, .17]	0.00<br>(0.94)<br>[-.04, .13]	-0.06*<br>(0.05)<br>[-.15, .01]	-0.01<br>(0.64)<br>[-.08, .08]	-0.01<br>(0.77)<br>[-.09, .08]	0.00<br>(0.88)<br>[-.10, .07]	-0.07*<br>(0.04)<br>[-.14, -.00]	-0.01<br>(0.78)<br>[-.08, .08]	1	
(14) Mother is married	0.05<br>(0.18)<br>[-.03, .14]	-0.02<br>(0.67)<br>[-.12, .05]	-0.01<br>(0.82)<br>[-.11, .06]	-0.06<br>(0.11)<br>[-.16, .01]	-0.04<br>(0.25)<br>[-.14, .03]	-0.04<br>(0.17)<br>[-.14, .02]	0.07*<br>(0.02)<br>[0.00, .13]	-0.01<br>(0.85)<br>[-.11, .06]	-0.04<br>(0.24)<br>[-.15, .02]	0.00<br>(0.98)<br>[-.08, .08]	0.14*<br>(0.00)<br>[.05, .21]	0.10*<br>(0.02)<br>[.01, .18]	-0.09*<br>(0.00)<br>[-.21, -.05]	1

Note. P-values in parentheses, 95% confidence intervals in brackets. * $p < .05$.

Table S3-2. Continuation of correlation matrix of child epigenetic indices and baseline covariates.

Variables	1	2	3	4	5	6	7	8	9	10	11	12	13
(1) Child DunedinPACE	1												
(2) Child Epigenetic-g_across	-0.06<br>(0.09)<br>[-.13, .01]	1											
(3) Child Epigenetic-g_separate	-0.10*<br>(0.01)<br>[-.17, -.03]	0.97*<br>(0.00)<br>[.97, .97]	1										
(4) Child PhenoAge Accel.	0.11*<br>(0.00)<br>[.04, .18]	0.01<br>(0.73)<br>[-.06, .09]	0.04<br>(0.33)<br>[-.04, .11]	1									
(5) Child GrimAge Accel.	0.18*<br>(0.00)<br>[.11, .25]	-0.04<br>(0.33)<br>[-.11, .04]	-0.04<br>(0.27)<br>[-.11, .03]	0.51*<br>(0.00)<br>[.45, .56]	1								
(6) Number of children	0.03<br>(0.45)<br>[-.04, .10]	-0.10*<br>(0.01)<br>[-.17, -.03]	-0.09*<br>(0.01)<br>[-.16, -.02]	-0.04<br>(0.31)<br>[-.11, .04]	-0.02<br>(0.61)<br>[-.09, .05]	1							
(7) Maternal Health	-0.08*<br>(0.03)<br>[-.15, -.00]	-0.02<br>(0.55)<br>[-.09, .05]	-0.01<br>(0.81)<br>[-.08, .06]	0.01<br>(0.74)<br>[-.06, .09]	0.06<br>(0.14)<br>[-.02, .13]	-0.08*<br>(0.01)<br>[-.18, -.03]	1						
(8) Maternal Depression	0.03<br>(0.44)<br>[-.04, .10]	0.05<br>(0.15)<br>[-.02, .13]	0.06<br>(0.08)<br>[-.01, .14]	0.07<br>(0.06)<br>[-.00, .14]	0.00<br>(0.96)<br>[-.07, .07]	0.06<br>(0.07)<br>[-.00, .15]	-0.19*<br>(0.00)<br>[-.26, -.12]	1					
(9) Mother is White	0.01<br>(0.74)<br>[-.06, .09]	0.04<br>(0.23)<br>[-.03, .12]	0.04<br>(0.24)<br>[-.03, .12]	-0.01<br>(0.81)<br>[-.08, .06]	0.04<br>(0.26)<br>[-.03, .11]	0.01<br>(0.75)<br>[-.06, .09]	0.00<br>(0.92)<br>[-.09, .05]	0.01<br>(0.68)<br>[-.07, .07]	1				
(10) Mother is Black	-0.10*<br>(0.01)<br>[-.17, -.03]	0.02<br>(0.65)<br>[-.06, .09]	0.03<br>(0.39)<br>[-.04, .11]	0.16*<br>(0.00)<br>[.09, .23]	0.11*<br>(0.00)<br>[.04, .18]	0.04<br>(0.24)<br>[-.07, .08]	0.07*<br>(0.04)<br>[.03, .18]	0.10*<br>(0.00)<br>[.02, .16]	-0.28*<br>(0.00)<br>[-.32, -.19]	1			
(11) Mother is Multiple Races	0.00<br>(0.98)<br>[-.07, .07]	-0.03<br>(0.35)<br>[-.12, .04]	-0.04<br>(0.27)<br>[-.11, .03]	-0.02<br>(0.66)<br>[-.09, .06]	-0.10*<br>(0.01)<br>[-.17, -.03]	0.02<br>(0.57)<br>[-.02, .12]	-0.02<br>(0.51)<br>[-.12, .02]	0.12*<br>(0.00)<br>[.08, .22]	-0.06*<br>(0.04)<br>[-.13, .01]	-0.16*<br>(0.00)<br>[-.23, -.09]	1		
(12) Mother is Other Race	0.07*<br>(0.05)<br>[.00, .15]	-0.06<br>(0.11)<br>[-.13, .01]	-0.06<br>(0.11)<br>[-.13, .01]	-0.03<br>(0.49)<br>[-.10, .05]	0.00<br>(0.90)<br>[-.07, .08]	0.10*<br>(0.00)<br>[.00, .14]	-0.07*<br>(0.03)<br>[-.21, -.07]	0.04<br>(0.17)<br>[-.02, .13]	-0.07*<br>(0.04)<br>[-.13, .02]	-0.17*<br>(0.00)<br>[-.22, -.08]	-0.04<br>(0.22)<br>[-0.11, .04]	1	
(13) Mother is Hispanic	0.06<br>(0.08)<br>[-.01, .14]	-0.01<br>(0.81)<br>[-.08, .06]	-0.02<br>(0.57)<br>[-.09, .05]	-0.15*<br>(0.00)<br>[-.22, -.08]	-0.10*<br>(0.01)<br>[-.17, -.03]	-0.09*<br>(0.00)<br>[-.13, -.00]	-0.02<br>(0.58)<br>[-.08, .06]	-0.18*<br>(0.00)<br>[-.24, -.10]	-0.28*<br>(0.00)<br>[-.34, -.21]	-0.70*<br>(0.00)<br>[-.76, -.69]	-0.16*<br>(0.00)<br>[-.24, -.10]	-0.17*<br>(0.00)<br>[-.23, -.09]	1

Note. P-values in parentheses, 95% confidence intervals in brackets. * $p < .05$

Table S4.

Weighted impacts of the cash gift on maternal and child epigenetic profiles in sensitivity analysis weighting for non-response.

	Unweighted Estimates (Table 1)		Inverse Probability of Treatment Weights - Average Treatment Effect on the Treated		Non-Response Weights - Average Treatment Effect	
	Effect Size (95% CI)	P-value	Effect Size (95% CI)	Unadjusted P-value	Effect Size (95% CI)	Unadjusted P-value
Maternal Epigenetic Profiles						
DunedinPACE	0.00 (-0.15 to 0.15)	0.981	-0.05 (-0.21 to 0.11)	0.519	0.00 (-0.15 to 0.15)	0.999
Epigenetic-g_ separate[a]	0.00 (-0.15 to 0.14)	0.978	-0.02 (-0.17 to 0.12)	0.744	-0.01 (-0.15 to 0.14)	0.901
Epigenetic-g_ across[a]	0.01 (-0.14 to 0.15)	0.925	-0.02 (-0.17 to 0.13)	0.785	0.00 (-0.14 to 0.15)	0.989
Child Epigenetic Profiles						
DunedinPACE	-0.17* (-0.32 to -0.01)	0.037	-0.19* (-0.36 to -0.03)	0.023	-0.17* (-0.32 to -0.01)	0.032
Epigenetic-*g*_ separate [a]	-0.15 (-0.31 to 0.02)	0.077	-0.11 (-0.28 to 0.05)	0.175	-0.14 (-0.30 to 0.02)	0.089
Epigenetic-*g*_ across[a]	-0.19* (-0.35 to -0.04)	0.016	-0.16 (-0.32 to 0.00)	0.053	-0.18* (-0.34 to -0.03)	0.021

Note. A respondent is defined as contributing a saliva sample for epigenetic analyses. Column 1 displays unweighted results which mimic those reported in Table 1. Column 2 displays results using Inverse Probability of Treatment Weights that balance the baseline characteristics of control group respondents to treatment group respondents at age-4 and estimate the Average Treatment Effect on the Treated. Column 3 displays results using Non-Response Weights that balance the baseline characteristics of respondents at age-4 to the full sample and estimate the Average Treatment Effect. [a]One robustness check differentiated Epigenetic-*g* computed separately for maternal and child DNAm (*i.e.,* Epigenetic-*g*_separate) versus Epigenetic-*g* computed across maternal and child DNAm together (*i.e.,* Epigenetic-*g*_across). *$p<0.05$.

Table S5.

Standardized parameter estimates from a model where child EEG regional absolute power measures were regressed on epigenetic indices.

	DunedinPACE	Epigenetic-*g* separate	Epigenetic-*g*_across
Frontal			
Theta	-0.03 (0.04)	0.03 (0.04)	0.03 (0.04)
Alpha	-0.05 (0.03)	0.02 (0.04)	0.02 (0.04)
Beta	-0.01 (0.04)	-0.04 (0.06)	-0.04 (0.06)
Gamma	-0.01 (0.04)	-0.04 (0.06)	-0.04 (0.06)
Temporal			
Theta	-0.02 (0.04)	0.04 (0.03)	0.04 (0.03)
Alpha	-0.04 (0.04)	0.01 (0.03)	0.02 (0.03)
Beta	0.01 (0.04)	-0.04 (0.04)	-0.05 (0.04)
Gamma	0.02 (0.04)	-0.05 (0.04)	-0.06 (0.04)
Occipital			
Theta	-0.01 (0.04)	0.02 (0.04)	0.03 (0.04)
Alpha	-0.02 (0.04)	0.02 (0.04)	0.03 (0.03)
Beta	-0.03 (0.04)	-0.04 (0.04)	-0.04 (0.04)
Gamma	0.01 (0.03)	-0.06 (0.05)	-0.06 (0.05)
Parietal			
Theta	0.00 (0.04)	-0.03 (0.04)	-0.02 (0.04)
Alpha	-0.01 (0.04)	0.01 (0.04)	0.02 (0.03)
Beta	-0.02 (0.04)	-0.03 (0.04)	-0.03 (0.04)
Gamma	0.04 (0.04)	-0.06 (0.04)	-0.05 (0.04)
Central			
Theta	-0.01 (0.04)	0.02 (0.03)	0.01 (0.03)
Alpha	-0.03 (0.04)	0.04 (0.04)	0.04 (0.04)
Beta	-0.02 (0.04)	0.02 (0.04)	0.01 (0.04)
Gamma	-0.00 (0.03)	-0.04 (0.04)	-0.04 (0.04)

Note. N=588. Models adjust for child sex, child age at the time of the assessment (in months), and the number of useable epochs. All variables were standardized to the full sample prior to analyses, so coefficients can be interpreted as standardized betas. Robust standard errors in parentheses. Epigenetic profiles were residualized for technical factors prior to regression analyses (cell composition, slide, and array). Sample is restricted to those with sufficient EEG data. Regional electrodes were preregistered as the following: **frontal** (20, 12, 5, 118, 24, 19, 11, 4, 124, 27, 23,18, 16, 10, 3, 123), **central** (6, 112, 105, 87, 79, 54, 37, 30, 13, 106, 80, 55, 31, 7, Cz), **parietal** (53, 61, 62, 78, 86, 52, 60, 67, 72, 77, 85, 92), **temporal** (40, 46, 39, 45, 50,109, 102, 115, 108, 101), and **occipital** (66, 71, 76, 84, 70, 75, 83). [a]One robustness check differentiated Epigenetic-*g* computed separately for maternal and child DNAm (*i.e.,* Epigenetic-*g*_separate) versus Epigenetic-*g* computed across maternal and child DNAm together (*i.e.,* Epigenetic-*g*_across). *p<0.05.

Table S6. Interaction of the cash gift and child epigenetic profiles on health, cognition, and relative EEG power.

	Health		Cognition			Relative EEG Power				Behavior	
	**BMI**	**Overall Health**	**Executive Function**	**Receptive Vocabulary Monolinguals**	**Receptive Vocabulary Bilinguals**	**Theta**	**Alpha**	**Beta**	**Gamma**	**Total Behavior Problems Index**	**Attn/Impulse Control Subscale**
DunedinPACE	0.02 (0.05) [0.694]	-0.02 (0.05) [0.659]	-0.02 (0.05) [0.640]	-0.03 (0.07) [0.665]	-0.06 (0.09) [0.560]	0.02 (0.07) [0.721]	-0.04 (0.06) [0.440]	0.01 (0.05) [0.849]	0.04 (0.05) [0.395]	-0.03 (0.05) [0.602]	-0.04 (0.05) [0.457]
Group	0.04 (0.08) [0.604]	-0.11 (0.08) [0.159]	0.11 (0.08) [0.165]	0.10 (0.11) [0.370]	-0.15 (0.12) [0.206]	-0.10 (0.09) [0.246]	0.16 (0.08) [0.062]	-0.06 (0.09) [0.462]	-0.07 (0.08) [0.419]	-0.00 (0.07) [0.971]	0.05 (0.07) [0.466]
Group* DunedinPACE	-0.06 (0.07) [0.436]	-0.07 (0.08) [0.384]	0.07 (0.08) [0.369]	0.05 (0.11) [0.629]	0.01 (0.14) [0.923]	0.02 (0.09) [0.823]	0.02 (0.08) [0.799]	-0.04 (0.08) [0.647]	-0.09 (0.07) [0.231]	0.04 (0.07) [0.522]	0.14 (0.08) [0.067]
Epigenetic-g_ separate			0.00 (0.06) [0.972]	0.03(0.08) [0.670]	-0.06(0.10) [0.538]	0.05 (0.06) [0.365]	0.00 (0.05) [0.983]	-0.11 (0.06) [0.054]	-0.07 (0.05) [0.176]	0.04(0.05) [0.487]	-0.01(0.05) [0.895]
Group			0.11 (0.08) [0.162]	0.10 (0.11) [0.386]	-0.14 (0.12) [0.272]	-0.10 (0.09) [0.238]	0.16 (0.08) [0.052]	-0.07 (0.09) [0.418]	-0.07 (0.08) [0.374]	0.00 (0.07) [0.968]	0.04 (0.07) [0.547]
Group* Epigenetic-g_ separate			0.03 (0.09) [0.729]	-0.08 (0.11) [0.468]	0.13 (0.15) [0.365]	-0.07 (0.09) [0.440]	0.03 (0.08) [0.649]	0.07 (0.09) [0.428]	0.04 (0.08) [0.613]	0.00(0.07) [0.959]	-0.03(0.06) [0.671]
Epigenetic-g_ across			0.01 (0.06) [0.920]	0.04 (0.08) [0.598]	-0.05 (0.09) [0.561]	0.06 (0.06) [0.277]	-0.01 (0.05) [0.811]	-0.11* (0.05) [0.047]	-0.06 (0.05) [0.218]	0.04 (0.05) [0.482]	-0.01 (0.05) [0.823]
Group			0.11 (0.08) [0.156]	0.10 (0.11) [0.383]	-0.14 (0.12) [0.263]	-0.10 (0.09) [0.245]	0.16 (0.08) [0.052]	-0.07 (0.09) [0.394]	-0.08 (0.08) [0.355]	0.01 (0.07) [0.939]	0.04 (0.07) [0.558]
Group* Epigenetic-g_ across			0.03 (0.09) [0.749]	-0.08 (0.11) [0.474]	0.17 (0.14) [0.249]	-0.07 (0.09) [0.388]	0.05 (0.08) [0.481]	0.06 (0.09) [0.502]	0.02 (0.08) [0.835]	0.01 (0.07) [0.871]	-0.02 (0.07) [0.742]

Note. Robust standard errors in parentheses, p-values in brackets. “Group" refers to the main effect of being randomly assigned to the high-cash gift group at baseline. Models adjust for child sex, age (in months), baseline covariates, and assessor ID. Continuous variables were standardized to the full sample prior to analyses. Coefficients can be interpreted as standardized betas. Epigenetic scores were residualized for technical factors prior to regression analyses, i.e., cell composition, slide, and array. Brain activity models further adjust for the number of useable epochs and are restricted to those with sufficient EEG data. *p<0.05.

Table S7. Interaction of the cash gift and child epigenetic profiles on health, cognition, and relative EEG power.

	Health		Cognition			Relative EEG Power				Behavior	
	**BMI**	**Overall Health**	**Executive Function**	**Receptive Vocabulary Monolinguals**	**Receptive Vocabulary Bilinguals**	**Theta**	**Alpha**	**Beta**	**Gamma**	**Total Behavior Problems Index**	**Attn/Impulse Control Subscale**
PhenoAge Accel	-0.02 (0.04)<br>[0.705]	-0.01 (0.06)<br>[0.895]	-0.08 (0.05)<br>[0.133]	-0.11 (0.08)<br>[0.176]	-0.01 (0.13)<br>[0.968]	-0.10 (0.07)<br>[0.178]	0.08 (0.06)<br>[0.222]	0.06 (0.06)<br>[0.306]	0.02 (0.06)<br>[0.734]	0.01 (0.05)<br>[0.897]	-0.10 (0.05)<br>[0.060]
Group	0.04 (0.08)<br>[0.604]	-0.09 (0.08)<br>[0.223]	0.12 (0.08)<br>[0.129]	0.09 (0.11)<br>[0.433]	-0.14 (0.13)<br>[0.293]	-0.10 (0.09)<br>0.262	0.16 (0.08)<br>[0.064]	-0.07 (0.09)<br>[0.440]	-0.07 (0.08)<br>[0.400]	-0.00 (0.07)<br>[0.987]	0.05 (0.07)<br>[0.443]
Group* PhenoAge Accel	0.04 (0.08)<br>[0.628]	-0.04 (0.08)<br>[0.573]	-0.02 (0.08)<br>[0.755]	0.14(0.09)<br>[0.143]	-0.05(0.17)<br>[0.776]	0.03 (0.10)<br>0.746	-0.01 (0.09)<br>[0.909]	-0.05 (0.10)<br>[0.580]	-0.02 (0.10)<br>[0.805]	-0.03(0.07)<br>[0.689]	0.01(0.08)<br>[0.862]
GrimAge Accel	0.11 (0.05)<br>[0.053]	0.04 (0.05)<br>[0.418]	-0.03 (0.05)<br>[0.625]	-0.10 (0.07)<br>[0.124]	-0.17 (0.11)<br>[0.101]	-0.04 (0.07)<br>0.579	-0.00 (0.06)<br>[0.951]	0.05 (0.06)<br>[0.347]	0.08 (0.06)<br>[0.173]	0.03 (0.05)<br>[0.561]	-0.11* (0.05)<br>[0.022]
Group	0.04 (0.08)<br>[0.608]	-0.10 (0.08)<br>[0.209]	0.11 (0.08)<br>[0.168]	0.10 (0.11)<br>[0.366]	-0.11 (0.12)<br>[0.366]	-0.11 (0.09)<br>0.229	0.17* (0.08)<br>[0.049]	-0.07 (0.09)<br>[0.441]	-0.08 (0.08)<br>[0.369]	-0.00 (0.07)<br>[0.971]	0.05 (0.07)<br>[0.473]
Group* GrimAge Accel	-0.05 (0.08)<br>[0.565]	-0.17* (0.08)<br>[0.030]	-0.07 (0.07)<br>[0.335]	0.05(0.10)<br>[0.631]	0.25(0.15)<br>[0.095]	-0.03 (0.10)<br>[0.771]	0.09 (0.09)<br>[0.316]	-0.06 (0.09)<br>[0.477]	-0.11 (0.09)<br>[0.228]	-0.07(0.07)<br>[0.305]	0.01(0.07)<br>[0.916]

Note. Robust standard errors in parentheses, p-values in brackets. 'Treatment group" refers to the main effect of being randomly assigned to the high-cash gift group at baseline. Models adjust for child sex, age (in months), baseline covariates, and assessor ID. Continuous variables were standardized to the full sample prior to analyses. Coefficients can be interpreted as standardized betas. Epigenetic scores were residualized for technical factors prior to regression analyses, i.e., cell composition, slide, and array. Brain activity models further adjust for the number of useable epochs and are restricted to those with sufficient EEG data. *$*p<0.05$.*

Table S8. Correlation matrix of maternal epigenetic indices and baseline covariates.

Variables	1	2	3	4	5	6	7	8	9	10	11	12	13	14	15	16	17	18	19	20	21	22
(1) Mother DunedinPACE	1																					
(2) Mother Epigenetic-g_across	.04	1																				
(3) Mother Epigenetic-g_separate	.05	.98*	1																			
(4) Mother PhenoAge Accel.	.17*	.11*	.11*	1																		
(5) Mother GrimAge Accel.	.19*	.02	.05	.25*	1																	
(6) Number of children	.09*	.07*	.11*	.01	.03	1																
(7) Maternal health	-.02	-.01	.00	-.03	.00	-.08*	1															
(8) Maternal depression	-.01	.01	.01	-.01	.12*	.06	-.19*	1														
(9) Completed H.S.	.00	-.09*	-.10*	-.04	-.01	-.03	.00	.01	1													
(10) Mother is White	-.05	.10*	.09*	-.01	.09*	.01	.00	.01	.00	1												
(11) Mother is Black	-.05	-.15*	-.16*	-.02	.13*	.04	.07*	.10*	.15*	-.28*	1											
(12) Mother is Multiple Races	.06	.04	.04	.01	.08*	.02	-.02	.12*	.01	-.06*	-.16*	1										
(13) Mother is Other Race	.03	-.02	-.01	.01	.09*	.10*	-.07*	.04	-.02	-.07*	-.17*	-.04	1									
(14) Mother is Hispanic	.05	.09*	.09*	.00	-.24*	-.09*	-.02	-.18*	-.14*	-.28*	-.70*	-.16*	-.17*	1								
(15) Mother is married	.01	.04	.04	.03	-.14*	.18*	-.04	-.10*	-.09*	.02	-.20*	-.06	.03	.20*	1							
(16) Avg cigs per week during pregnancy	.03	.08*	.09*	-.04	.20*	.16*	-.07*	.13*	-.01	.30*	-.07*	.08*	.07*	-.17*	-.04	1						
(17) Avg drinks per week during pregnancy	-.04	-.03	-.03	-.04	.02	-.04	.03	.00	.00	.13*	-.05	.00	-.01	-.03	.00	.19*	1					
(18) Household Income	-.01	.02	.02	.00	-.07	.00	.00	-.08*	-.07*	.08*	-.16*	-.03	.07*	.10*	.14*	-.03	.01	1				
(19) Household Net Worth	-.05	.00	.00	.03	.05	-.03	-.04	-.03	-.01	.00	.00	-.02	.02	.00	.00	-.03	.00	.16*	1			
(20) Child is female	-.01	.00	-.01	-.03	-.04	-.12*	.03	-.01	.00	-.01	.04	-.05	-.04	.01	-.04	.01	.01	.01	-.02	1		
(21) Child Birthweight	.05	.11*	.12*	.00	-.09*	.09*	.02	-.09*	-.06*	.03	-.17*	.01	.04	.13*	.07*	-.04	-.01	.03	-.06	-.16*	1	
(22) Child Gestational Age	-.02	.01	.00	-.02	-.05	-.15*	.04	-.05	-.01	-.02	-.06*	-.02	-.02	.10*	-.01	-.13*	.00	.03	-.04	.02	.38*	1

Note. * $p<0.05$.
