# Supplementary material for: Effects of a randomized controlled trial of unconditional cash transfers on epigenetic measures of ageing and cognition in children and mothers
Source: Nat Hum Behav. 2026 Sep 8;10(10):2002–15. doi: 10.1038/s41562-026-02568-4 (PMC13683044; doi:10.1038/s41562-026-02568-4)
Supplement: Supplementary file 2 [file 41562_2026_2568_MOESM2_ESM.pdf]

nature portfolio

Corresponding author(s): Raffington, Harden, Noble

Last updated by author(s): Aug 12, 2025

Reporting Summary

Nature Portfolio wishes to improve the reproducibility of the work that we publish. This form provides structure for consistency and transparency in reporting. For further information on Nature Portfolio policies, see our Editorial Policies and the Editorial Policy Checklist.

Statistics

For all statistical analyses, confirm that the following items are present in the figure legend, table legend, main text, or Methods section.

n/a	Confirmed	
☐	☒	The exact sample size (n) for each experimental group/condition, given as a discrete number and unit of measurement
☐	☒	A statement on whether measurements were taken from distinct samples or whether the same sample was measured repeatedly
☐	☒	The statistical test(s) used AND whether they are one- or two-sided<br>*Only common tests should be described solely by name; describe more complex techniques in the Methods section.*
☐	☒	A description of all covariates tested
☐	☒	A description of any assumptions or corrections, such as tests of normality and adjustment for multiple comparisons
☐	☒	A full description of the statistical parameters including central tendency (e.g. means) or other basic estimates (e.g. regression coefficient) AND variation (e.g. standard deviation) or associated estimates of uncertainty (e.g. confidence intervals)
☐	☒	For null hypothesis testing, the test statistic (e.g. F, t, r) with confidence intervals, effect sizes, degrees of freedom and P value noted<br>*Give P values as exact values whenever suitable.*
☒	☐	For Bayesian analysis, information on the choice of priors and Markov chain Monte Carlo settings
☒	☐	For hierarchical and complex designs, identification of the appropriate level for tests and full reporting of outcomes
☐	☒	Estimates of effect sizes (e.g. Cohen's d, Pearson's r), indicating how they were calculated

Our web collection on statistics for biologists contains articles on many of the points above.

Software and code

Policy information about availability of computer code

Data collection	Not applicable
Data analysis	R and Stata software

For manuscripts utilizing custom algorithms or software that are central to the research but not yet described in published literature, software must be made available to editors and reviewers. We strongly encourage code deposition in a community repository (e.g. GitHub). See the Nature Portfolio guidelines for submitting code & software for further information.

Data

Policy information about availability of data

All manuscripts must include a data availability statement. This statement should provide the following information, where applicable:

- Accession codes, unique identifiers, or web links for publicly available datasets
- A description of any restrictions on data availability
- For clinical datasets or third party data, please ensure that the statement adheres to our policy

Summarized data, except for child chronological age due to HIPPA data sharing restrictions, and documentation is publicly available at the Inter-university Consortium for Political and Social Research (OPEN ICPSR; ID159422; https://www.openicpsr.org/openicpsr/project/159422/version/V8/view).

Research involving human participants, their data, or biological material

Policy information about studies with human participants or human data. See also policy information about sex, gender (identity/presentation), and sexual orientation and race, ethnicity and racism.

Reporting on sex and gender	High-cash and low-cash gift groups balanced for child sex (50% female). Only mothers amongst adults. Statistical models adjust for child sex.
Reporting on race, ethnicity, or other socially relevant groupings	Mothers self-identified as 40% Black, 40% Latinx, and 20% "other" or "white" or "Asian". Covariates from the baseline survey include: maternal characteristics (age, education, race and ethnicity, marital status, general health, depressive symptoms, and cigarette and alcohol consumption during pregnancy), household characteristics (number of children born to mother, number of adults in the household, father living with the mother, household income, and household net worth), and infant birth characteristics (weight at birth and gestational age).
Population characteristics	To be eligible, mothers' self-reported income in the prior calendar year had to fall below the U.S. federal poverty threshold for their family size. Additional study inclusion criteria were (1) the mother was of legal age for informed consent; (2) the infant was the product of a singleton pregnancy and admitted to the newborn nursery (not intensive care); (3) the mother was residing in the state of recruitment; (4) the mother indicated that she is not "highly likely" to move to a different state or country in the next 12 months; (5) the infant was discharged in the custody of the mother; and (6) the mother spoke English or Spanish.
Recruitment	Baby's First Years is a randomized controlled trial of cash transfers in early childhood in the United States 1. Launched in 2018, the project recruited 1,000 mothers and their newborn infants in 12 birth hospitals across four metropolitan areas in the US (New York City, the greater New Orleans metropolitan area, the greater Omaha metropolitan area, and the Twin Cities of Minneapolis and St. Paul). Shortly after giving birth, mothers were randomized to receive an unconditional monthly cash transfer of either $333/month ("high-cash group", n=400) or $20/month ("low-cash group", n=600), initially for the first 40 months of the child's life, and subsequently extended to the first 76 months of the child's life.
Ethics oversight	This research complies with all relevant ethical regulations. All study procedures were approved by the Institutional Review Boards of Teachers College, Columbia University (protocol #18-210), and the New York State Psychiatric Institute (protocol #7606).

Note that full information on the approval of the study protocol must also be provided in the manuscript.

Field-specific reporting

Please select the one below that is the best fit for your research. If you are not sure, read the appropriate sections before making your selection.

☐ Life sciences ☒ Behavioural & social sciences ☐ Ecological, evolutionary & environmental sciences

For a reference copy of the document with all sections, see nature.com/documents/nr-reporting-summary-flat.pdf

Behavioural & social sciences study design

All studies must disclose on these points even when the disclosure is negative.

Study description	Quantitative experimental
Research sample	Mothers with low incomes in the US. To be eligible, mothers' self-reported income in the prior calendar year had to fall below the U.S. federal poverty threshold for their family size.
Sampling strategy	Randomized controlled trial. During the age-4 university visit (which occurred July 2022 - August 2023), all mothers were invited to contribute saliva from themselves and their children to examine DNA methylation as an optional extension to the study.
Data collection	Mother and child saliva samples (2 mL) were collected using ORAcollect•Dx (OCD-100/100A, DNA Genotek Inc., Ontario, Canada). Saliva samples were stored in Oragene kits that release a DNA stabilizing buffer at room temperature.
Timing	July 2022 - August 2023
Data exclusions	DNA methylation data exclusion were pre-registered. CpG probes were excluded if 1) they had a detection of p > 0.01, 2) had fewer than 4 beads in more than 5% of the samples, or 3) had a success rate <.95 across all samples 9. Probes on the X or Y chromosome will not be excluded for the analyses 10. Samples were excluded if they 1) showed low intensity probes as indicated by the log of average methylation <9 and 2) their detection p is > 0.01 in > 10% of their probes.
Non-participation	N=220 participaning families declined to participate in saliva collection. See CONSORT Fig S1
Randomization	Randomized to high-cash and low-cash groups

Reporting for specific materials, systems and methods

We require information from authors about some types of materials, experimental systems and methods used in many studies. Here, indicate whether each material, system or method listed is relevant to your study. If you are not sure if a list item applies to your research, read the appropriate section before selecting a response.

Materials & experimental systems

n/a	Involved in the study
☒	☐ Antibodies
☒	☐ Eukaryotic cell lines
☒	☐ Palaeontology and archaeology
☒	☐ Animals and other organisms
☐	☒ Clinical data
☒	☐ Dual use research of concern
☒	☐ Plants

Methods

n/a	Involved in the study
☒	☐ ChIP-seq
☒	☐ Flow cytometry
☒	☐ MRI-based neuroimaging

Clinical data

Policy information about clinical studies

All manuscripts should comply with the ICMJE guidelines for publication of clinical research and a completed CONSORT checklist must be included with all submissions.

Clinical trial registration	NCT03593356
Study protocol	https://clinicaltrials.gov/study/NCT03593356
Data collection	During the age-4 university visit (which occurred July 2022 - August 2023), all mothers were invited to contribute saliva from themselves and their children to examine DNA methylation as an optional extension to the study.
Outcomes	We designated DNAm measures of epigenetic pace of aging (DunedinPACE) and Epigenetic-g as primary outcomes of interest, and two other epigenetic clocks (GrimAge and PhenoAge Acceleration) as secondary outcomes of interest, in both mothers and children, prior to commencing the age-4 data collection (clinical trial identifier NCT03593356). We also publicly preregistered a detailed data analysis plan after data collection had concluded, but prior to the generation of epigenetic data (https://osf.io/8grhj).

Plants

Seed stocks	*Report on the source of all seed stocks or other plant material used. If applicable, state the seed stock centre and catalogue number. If plant specimens were collected from the field, describe the collection location, date and sampling procedures.*
Novel plant genotypes	*Describe the methods by which all novel plant genotypes were produced. This includes those generated by transgenic approaches, gene editing, chemical/radiation-based mutagenesis and hybridization. For transgenic lines, describe the transformation method, the number of independent lines analyzed and the generation upon which experiments were performed. For gene-edited lines, describe the editor used, the endogenous sequence targeted for editing, the targeting guide RNA sequence (if applicable) and how the editor was applied.*
Authentication	*Describe any authentication procedures for each seed stock used or novel genotype generated. Describe any experiments used to assess the effect of a mutation and, where applicable, how potential secondary effects (e.g. second site T-DNA insertions, mosiacism, off-target gene editing) were examined.*
